# Thioester‐containing proteins in the tsetse fly (*Glossina*) and their response to trypanosome infection

**DOI:** 10.1111/imb.12382

**Published:** 2018-03-12

**Authors:** I. Matetovici, J. Van Den Abbeele

**Affiliations:** ^1^ Unit of Veterinary Protozoology, Department of Biomedical Sciences Institute of Tropical Medicine Antwerp (ITM) Antwerp Belgium

**Keywords:** thioester‐containing proteins, tsetse fly, trypanosome, innate immunity, vector–parasite interaction

## Abstract

Thioester‐containing proteins (TEPs) are conserved proteins with a role in innate immune immunity. In the current study, we characterized the TEP family in the genome of six tsetse fly species (*Glossina* spp.). Tsetse flies are the biological vectors of several African trypanosomes, which cause sleeping sickness in humans or nagana in livestock. The analysis of the tsetse TEP sequences revealed information about their structure, evolutionary relationships and expression profiles under both normal and trypanosome infection conditions. Phylogenetic analysis of the family showed that tsetse flies harbour a genomic expansion of specific TEPs that are not found in other dipterans. We found a general expression of all TEP genes in the alimentary tract, mouthparts and salivary glands. *Glossina morsitans* and *Glossina palpalis* TEP genes display a tissue‐specific expression pattern with some that are markedly up‐regulated when the fly is infected with the trypanosome parasite. A different TEP response was observed to infection with *Trypanosoma brucei* compared to *Trypanosoma congolense*, indicating that the tsetse TEP response is trypanosome‐specific. These findings are suggestive for the involvement of the TEP family in tsetse innate immunity, with a possible role in the control of the trypanosome parasite.

## Introduction

Being exposed to a range of pathogenic organisms, insects rely solely on innate immune responses that serve as the first line of defence, protecting them through mechanisms that can be activated rapidly upon recognition of a foreign threat. An immune response is activated when pattern recognition receptors (PRRs) detect pathogen‐associated molecules as ‘nonself’ and initiate a complex signal transduction cascade that finally leads to the production of specific effector molecules to control the microbial threat. PRRs include peptidoglycan‐recognition proteins and Gram‐negative‐binding proteins that detect Gram+/Gram– bacteria as well as fungi.

In addition to PRRs insects are equipped with secreted recognition molecules, opsonins, such as the thioester‐containing proteins (TEPs; Blandin & Levashina, 2004). These are characterized by a highly reactive thioester motif that can bind covalently to pathogen target molecule. TEPs are a large protein family divided into four subfamilies: (1) vertebrate complement factors (C3–C5) and C3‐like proteins, (2) alpha‐2‐macroglobulin (A_2_M), (3) insect TEP (iTEP) and (4) macroglobulin complement‐related (MCR) proteins (Blandin & Levashina, 2004; Palmer & Jiggins, 2015). In insects, these proteins have mostly been studied in mosquitoes and fruit flies whose genomes encode for only two TEP subfamilies, iTEPs and MCRs. In the malaria vector *Anopheles gambiae*, TEP1 is involved in opsonization of various pathogens including *Plasmodium* spp. (Levashina *et al*., 2001; Blandin *et al*., 2008; Yassine *et al*., 2012), and was demonstrated to play a key role in the removal of defective apoptotic sperm cells during spermatogenesis (Pompon & Levashina, 2015). *Aedes aegypti* macroglobulin complement‐related factor (*Ae. aegypti*_MCR) was shown to possess antiviral activity against dengue virus infection by induction of antimicrobial peptides (Xiao *et al*., 2014). In *Drosophila melanogaster*, TEP molecules display immuno‐modulatory responses to various pathogens, such as bacteria (Lagueux *et al*., 2000; Stroschein‐Stevenson *et al*., 2006; Igboin *et al*., 2011; Dostalova *et al*., 2017; Shokal & Eleftherianos, 2017), fungi (Stroschein‐Stevenson *et al*., 2006; Dostalova *et al*., 2017), nematodes (Arefin *et al*., 2014; Castillo *et al*., 2015) and parasitoids (Wertheim *et al*., 2005; Dostalova *et al*., 2017).

Besides the studies in mosquitoes and fruit flies there are only a handful of reports in other insects. Differential expression following microbial or parasitic infection has been reported for the house fly (Sackton *et al*., 2017) and for bumble bee workers (Erler *et al*., 2011). On the contrary, in the case of the tobacco hornworm (Gunaratna & Jiang, 2013) and the silkworm (Zhao *et al*., 2012), small or no changes were noted after microbial infection. Furthermore, in the honey bee genome one member of the A_2_M subfamily was identified, suggesting that the complement‐like system may not be broadly conserved in insects (Palmer & Jiggins, 2015).

In the tsetse fly (*Glossina* spp.) we have recently demonstrated an increased expression of some TEPs in *Trypanosoma brucei*‐infected salivary glands (Matetovici *et al*., 2016), which suggests a possible role of this protein family in controlling the parasite population in this micro‐environment. Tsetse flies (*Glossina* spp.) are obligate blood‐feeding insects and the exclusive biological vectors of different species of African trypanosomes in sub‐Saharan Africa, protozoan parasites that cause devastating diseases in humans [human African trypanosomiasis (HAT) – sleeping sickness; caused by *T. brucei complex*] and livestock [animal African trypanosomiasis (AAT) – nagana; mainly caused by *Trypanosoma congolense*, *Trypanosoma vivax*]. The fly ingests the trypanosomes through feeding on an infected host. Once ingested, the parasite goes through a complex developmental cycle in the fly's alimentary tract and mouthparts/salivary glands, depending on the trypanosome species (Rotureau & Van Den Abbeele, 2013), to achieve the final metacyclic infective state.

There are 31 species and subspecies of tsetse flies (*Glossina*) that are further subdivided into three groups (subgenera) – the Fusca, Morsitans and Palpalis groups – based on morphological characteristics (primarily genitalia structure), areal distribution, habitat and molecular data (Gooding & Krafsur, 2005; Krafsur, 2009). The Morsitans group (subgenus *Glossina sensu stricto*) includes tsetse fly vectors of HAT and AAT mainly in east and central Africa such as *Glossina morsitans*, *Glossina pallidipes* and *Glossina austeni*. The Palpalis group (subgenus Nemorhina) includes the major vectors of HAT in west and central Africa such as *Glossina palpalis* and *Glossina fuscipes*. Species of the Fusca group (subgenus Austenina) like *Glossina brevipalpis* have no significant medical or economic importance.

Most tsetse fly genomics and functional molecular research is focused on *G. m. morsitans* with only a few studies addressing the other tsetse fly vectors in a comparative approach (International Glossina Genome Initiative, 2014; Zhao *et al*., 2015; Macharia *et al*., 2016) owing to the limited availability of high‐quality genomic information for these species. However, very recently the genomes of five tsetse fly species (beside *G. m. morsitans*) became publicly available, prompting us to perform a genomic and functional analysis of the tsetse thioester‐containing proteins family. We show that the tsetse fly genomes encode between six to eight TEP genes, with differences between the species. Phylogenetic analysis of the family reveals that tsetse flies harbour some specific TEPs that are not found in any other dipterans. *G. m. morsitans* and *G. palpalis gambiensis* TEP genes display a tissue‐specific expression pattern with some that are markedly up‐regulated when the fly is infected with the trypanosome parasite. Interestingly, a different TEP response was observed to infection with *T*. *brucei* compared to *T*. *congolense*, indicating that the tsetse TEP response is trypanosome‐specific. Taken together, our findings demonstrate a genomic expansion of tsetse fly‐specific TEPs and are suggestive for the involvement of the TEP family in tsetse innate immunity with a possible role in the specific control of the trypanosome parasite in the fly.

## Results

### Genomic organization of *G. m. morsitans* TEPs

**Figure 1:**
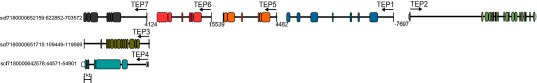
Thioester‐containing protein (TEP) gene organization in the *G. m. morsitans* genome. The coloured boxes denote exons, the lines in between are introns and the smaller white boxes represent untranslated regions; the arrows indicate the direction of transcription of each gene; the numbers between the genes represent the length in base pairs of the intergenic regions, with the negative number representing an overlapping region on the opposite strand. The dotted line around the *Gmm*_*TEP6*_ exon7 indicates the region obtained by cloning. The number before each locus represents the scaffold number (version GMOY1.1) followed by the start and end positions. Illustrator for Biological Sequences 1.02 (Liu *et al*., 2015) was used to construct the figure. *Gmm*, *Glossina morsitans morsitans*. [Colour figure can be viewed at http://wileyonlinelibrary.com]

**Figure 2:**
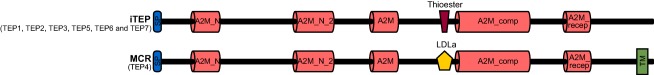
Thioester‐containing protein (TEP) smart domain architecture in *G. m. morsitans*. The functional modules of insect TEP (iTEP) and macroglobulin complement‐related (MCR) proteins were predicted with the smart and Pfam websites. SP, signal peptide for Gmm_TEP2, Gmm_TEP4, Gmm_TEP5, Gmm_TEP6 and Gmm_TEP7; A2M_N, A2M_N_2, A2M and A2M_comp, alpha‐2‐macroglobulin domains; A2M_recep, A2M receptor binding domain; LDLa, low‐density lipoprotein receptor domain class A; TM, transmembrane domain. Illustrator for Biological Sequences 1.02 (Liu *et al*., 2015) was used to construct the figure. Gmm, *Glossina morsitans morsitans*. [Colour figure can be viewed at http://wileyonlinelibrary.com]

To identify members of the thioester‐containing protein family, the *G. m. morsitans* (GmorY1) genome was screened with available *D. melanogaster* TEP sequences (Dmel_TEP1–TEP6). Seven genes coding for putative TEPs were found: *Gmm*_*TEP1* (GMOY010996), *Gmm*_*TEP2* (GMOY010998), *Gmm*_*TEP3* (GMOY008955), *Gmm*_*TEP4* (GMOY001989), *Gmm*_*TEP5*, *Gmm*_*TEP6* and *Gmm_TEP7*. Four of these TEP genes were previously annotated (International Glossina Genome Initiative, 2014) and three were newly identified. In fact, the latter were previously wrongly predicted as being part of the *TEP1* (GMOY010996) gene. Based upon these findings, we newly annotated these genes as *Gmm*_*TEP1*, *Gmm*_*TEP5* and *Gmm*_*TEP6* (Supporting Information Table S1). Owing to gaps in the genome assembly, only a partial coding sequence of 2386 bp was available for *Gmm*_*TEP6*. We determined the remaining part of the sequence (1821 bp) by PCR and Sanger sequencing (Supporting Information Figs S1 and S2). The *Gmm*_*TEP7* gene was identified *de novo* by querying the scaffold sequence (GmorY1:scf7180000652159) for open reading frames (ORFs) with the ORF finder program available from the National Center for Biotechnology Information (NCBI; Supporting Information Figs S1 and S2). Exon–intron structures were predicted using GenScan software and validated by visual inspection in Integrative Genomics Viewer software (Thorvaldsdottir *et al*., 2013) of tsetse fly RNA sequencing (RNA‐seq) mapped reads (Matetovici *et al*., 2016). The coordinates of the seven *G. m. morsitans* genes encoding thioester‐containing proteins are listed in Supporting Information Table S1. Five of the genes, *Gmm*_*TEP1*, *Gmm*_*TEP2*, *Gmm*_*TEP5*, *Gmm*_*TEP6* and *Gmm*_*TEP7*, are located on the same genomic scaffold spanning a region of over 80 kb, with *Gmm*_*TEP7*, *Gmm*_*TEP6*, *Gmm*_*TEP5* and *Gmm*_*TEP1* tandemly arrayed on one strand, whereas *Gmm*_*TEP2* is present on the opposite strand with an approximately 7.6 kb overlap with *Gmm*_*TEP1* (Fig. 1). This clustering may have resulted from a duplication‐inversion event, followed by recent tandem duplication. The other two genes, *Gmm*_*TEP3* and *Gmm*_*TEP4*, are found at different locations in the genome (Fig. 1). The gene length varies considerably between different family members, from 7 to 20 kb (Fig. 1 and Supporting Information Table S1). Furthermore, the presence of a putative signal peptide is predicted for Gmm_TEP2, Gmm_TEP4, Gmm_TEP5, Gmm_TEP6 and Gmm_TEP7, indicating that they are probably secreted. The functional domain architecture of the tsetse TEPs was predicted by the smart program (Fig. 2). Out of the seven TEPs, six contain a thioester motif, whereas Gmm_TEP4 displays a low‐density lipoprotein receptor domain class A (LDLa) instead. Moreover, four proteinase‐binding alpha‐2‐macroglobulin domains (A_2_M‐N, A_2_M‐N2, A_2_M and A_2_M_comp) and one alpha‐2‐macroglobulin receptor binding domain (A_2_M_recep) are present in all TEP sequences (Fig. 2). Gmm_TEP4 also contains a transmembrane domain.

Similar to *D. melanogaster* (Lagueux *et al*., 2000) three additional features are found to be characteristic for the *G. m. morsitans* TEPs: (1) a highly conserved region of about 40 amino acids residues harbouring the thioester motif (or LDLa in the case of Gmm_TEP4); (2) a variable region of approximatively 120 residues between the A_2_M‐N‐2 and A_2_M domain and (3) a cysteine signature in the C‐terminal part (Supporting Information Fig. S1).

### Genomic organization of *Glossina* species TEPs

**Figure 3:**
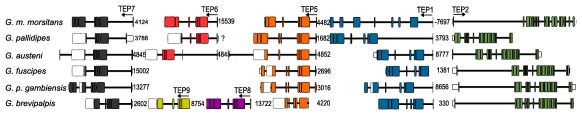
Genomic organization of thioester‐containing protein (TEP) genes in Glossinidae. The coloured boxes denote exons, the lines in between are introns and the smaller white boxes represent untranslated regions; the arrows indicate the direction of transcription of each gene; the numbers between the genes represent the length in base pairs of the intergenic regions, with the negative number representing an overlapping region on the opposite strand. ?, unknown. The dotted line around the *Gmm*_*TEP6*_exon7 indicates the region obtained by cloning. The white dotted boxes indicate gaps in the genome assembly. Gene characteristics are described in Supporting Information Table S1. Illustrator for Biological Sequences 1.02 (Liu *et al*., 2015) was used to construct the figure. *G*., *Glossina*. [Colour figure can be viewed at http://wileyonlinelibrary.com]

Recently, the genomes of five other tsetse fly species became available at VectorBase: *G*. *brevipalpis* (Fusca group), *G*. *fuscipes* and *G. p. gambiensis* (Palpalis group), *G*. *pallidipes* and *G*. *austeni* (Morsitans group). We screened these genomes using the *G. m. morsitans* homologue sequences. The identified and annotated TEP sequences are summarized in Supporting Information Table S1. Overall, the genomes of these five tsetse species have six to eight TEP genes, with *G*. *brevipalpis* having the highest and *G*. *fuscipes* the lowest number of genes. The genome organization is similar to the one described for *G. m. morsitans* with genes encoding for *TEP1*, *TEP2*, *TEP5*, *TEP6*, *TEP7* and *TEP8*, *TEP9* located on the same genomic scaffold (Fig. 3) and *TEP3* and *TEP4* at different locations. The *TEP6* gene is not present in the Palpalis group (*G. fuscipes* and *G. p. gambiensis*). Moreover, two copies of the *Gpg*_*TEP3* gene are found in the *G. p. gambiensis* genome. Two homologues, *Gb_TEP8* and *Gb_TEP9*, are present only in the Fusca group (*G. brevipalpis*; Fig. 3). Unfortunately, owing to gaps in the currently available genome assembly, only partial gene sequences for *TEP5*, *TEP6* and *TEP7* are available for most of the tsetse species. An iterative mapping and assembly of publicly available tsetse species‐specific RNA‐seq reads as an attempt to close the gaps was unsuccessful. Indeed, the C‐terminal of these three genes is highly conserved making it difficult to distinguish between the multi mapping reads (data not shown).

A potential signal peptide is predicted for the following proteins: TEP2 for the Palpalis group, TEP3 for all the species (except *G. p. gambiensis* GPPI047920), TEP4 for all species (except *G*. *pallidipes*), TEP5 for all species (except *G*. *brevipalpis*), TEP6 for *G*. *pallidipes* and *G. m. morsitans*, TEP7 for all species (except *G*. *brevipalpis*) and TEP8 for *G*. *brevipalpis* (Supporting Information Table S1).

### Phylogenetic analysis of tsetse fly TEPs

**Figure 4:**
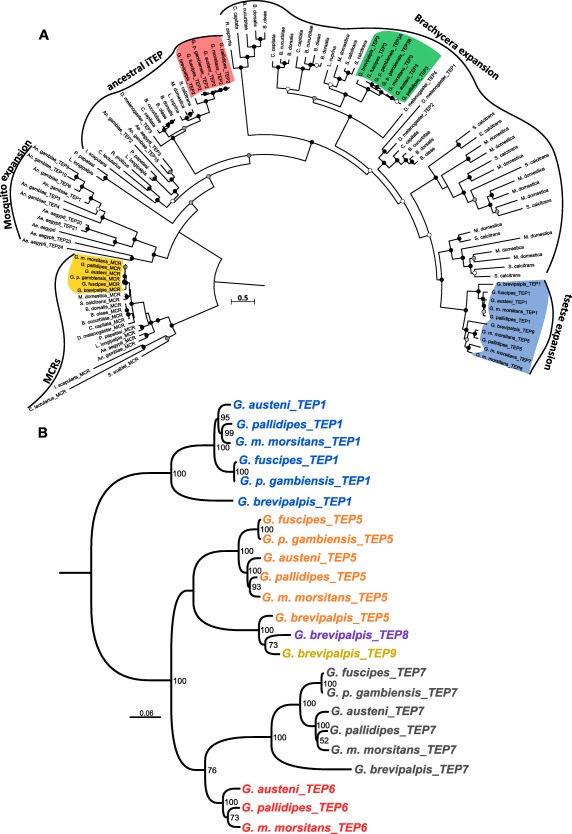
Phylogenetic analysis of tsetse fly thioester‐containing proteins (TEPs). (A) The amino acid sequence alignment was performed using the muscle algorithm and the phylogenetic tree was reconstructed using the maximum likelihood method with the LG + G + I + F evolutionary model, 100 bootstrap replications. Bootstrap values are symbolized with a coloured circle at every node as follows: black 90–100, grey 89–70 and white less than 69. Scale bar is substitutions per site. Database and accession number of each TEP sequence can be found in Supporting Information Table S2. (B) The nucleotide sequence alignment was performed using the muscle algorithm and the phylogenetic tree was reconstructed using the maximum likelihood method with the general time reversible (GTR) + G + I evolutionary model, 100 bootstrap replications. Scale bar is substitutions per site. Database and accession number of each TEP sequence can be found in Supporting Information Table S1. *Ae., Aedes; An., Anopheles; B., Bactrocera; C.,Ceratitis; D., Drosophila; G., Glossina; H., Harpegnathos; L., Lucilia; M., Musca; M., Megachile; N., Nasonia; O., Orussus; R., Rhagoletis; S., Stomoxys; T., Tribolium;* iTEP, insect TEP protein; MCRs, macroglobulin complement‐related proteins. [Colour figure can be viewed at http://wileyonlinelibrary.com]

The TEP amino acid sequences of the six tsetse fly species were aligned with known TEP sequences using the Muscle program. As described above, the tsetse fly genomes encode for TEP genes belonging to the subfamilies MCR and iTEP. Homologous sequences found in different dipterans or blood‐feeding arthropods were used in the construction of the phylogenetic tree (Supporting Information Table S2). Only complete sequences were maintained in the final alignment. The phylogenetic tree was constructed based on a 2115 amino acid alignment from 114 sequences using the maximum likelihood (ML) method and LG substitution model (Fig. 4A).

The MCR group comprises genes encoding for the macroglobulin complement related proteins. As expected, the TEP4 sequences (MCR) of the six tsetse species are grouped together with the other insect MCRs, forming a sister group to the *Stomoxys calcitrans* and *Musca domestica* clade.

The iTEP group includes the ‘ancestral’ iTEP group (Bou Aoun *et al*., 2011) as well as several different species‐specific expansion groups: mosquitoes, different fruit flies, house fly, stable fly and tsetse fly. The ‘ancestral’ iTEP group was already characterized in Bou Aoun *et al*., (2011), Palmer & Jiggins (2015) and Sekiguchi & Nonaka (2015). In order not to overload the tree, we included only sequences from dipterans and blood‐feeding arthropods. All six tsetse fly TEP2 orthologues are part of this group and cluster together with *D. melanogaster*_TEP3 and with the other Brachycera fly species. The TEP3–TEP9 tsetse sequences are clustered in a branch restricted to the Brachycera suborder, which is divided into three main groups. The first group contains sequences found in five species of the Tephritidae family (*Rhagoletis zephyria*, *Ceratitis capitata*, *Bactrocera cucurbitae*, *Bactrocera dorsalis* and *Bactrocera oleae*). The second group consists of *D. melanogaster_*TEP4, TEP3 in tsetse species (with two copies for *G. p. gambiensis*), one sequence for *M. domestica*, two sequences located in a tandem array for *S*. *calcitrans* and two paralogous sequences in a tandem array in four Tephritidae species. The third group contains *D. melanogaster_*TEP1 and _TEP2, one sequence for each of the four Tephritidae species and an increased expansion in Muscidae and Glossinidae. Here, the tsetse TEP1, TEP5, TEP6, TEP7, TEP8 and TEP9 are all grouped together and no orthologues were found in any of the analysed dipteran species.

To gain a better view on the evolution of the TEP family within the Glossinidae, a second phylogenetic tree was constructed. The tree included only the TEP sequences found in the tandem array specific to tsetse flies and was based on a 1700‐nucleotide alignment using the ML method with general time reversible (GTR) as the substitution model (Fig. 4B), representing the A_2_M_N and A_2_M_N_2 domains. Taking into consideration the phylogeny as described above (Fig. 4A), tsetse TEP5 and TEP7 were generated after the divergence of Glossinidae and Muscidae and before the split of the different Glossinidae groups. TEP8 and TEP9 most likely emerged from TEP5 duplication after the separation of the Fusca group, as these genes are found only in *G. brevipalpis* and absent in the other groups. Moreover, TEP6 was most likely duplicated from TEP7 in the Morsitans group after the divergence of the Palpalis group.

### Conservation of the CGEQ–thioester site

**Figure 5:**
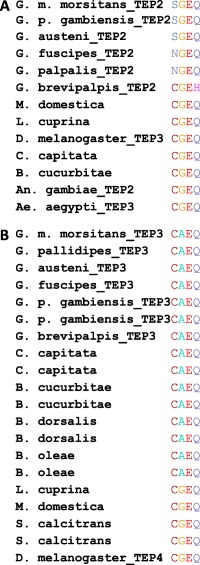
Sequence alignment of the canonical thioester site. (A) Orthologues of tsetse thioester‐containing protein 2 (TEP2; sequences that cluster in ‘ancestral iTEP’ Fig. 4A); (B) orthologues of tsetse TEP3 (sequences that cluster in Fig. 4A in the second group of the ‘Brachycera expansion’). *Ae., Aedes; An., Anopheles; B., Bactrocera; C.,Ceratitis; D., Drosophila; G., Glossina; L., Lucilia; M., Musca; S., Stomoxys;* iTEP, insect TEP. [Colour figure can be viewed at http://wileyonlinelibrary.com]

Specific to the thioester‐containing proteins is the CGEQ–thioester site that plays a key role in the formation of a covalent bond to microbial surfaces. Variation in this site is linked with the inability of the protein to bind to microbes. Sequence analysis of the six *Glossina* homologues (Fig. 5) with a thioester motif indicated that the canonical thioester motif is present (Supporting Information Fig. S1) in TEP1, TEP5, TEP6 and TEP7 whereas TEP2 and TEP3 showed variation from the canonical sequence (Fig. 5 and Supporting Information Fig. S1).

One type of variation identified in the TEP2 homologues is the replacement of cysteine (first position) with serine in species belonging to the Morsitans group and with asparagine in the Palpalis group. A second type of substitution in TEP2 is observed for the Fusca group, where glutamine (position four) is replaced by a histidine (Fig. 5A). Another substitution that is present in all tsetse TEP3 sequences is that of glycine (second position) by alanine. This type of substitution is also found in flies belonging to the Tephritidae family (*C. capitata*, *B. cucurbitae*, *B. dorsalis* and *B. oleae*; Fig. 5B) as well in vertebrate homologues like the mouse C4 (Nonaka *et al*., 1985) and cattle C4 (Ren *et al*., 1993).

### *G. m. morsitans* TEP expression in different tissues

**Figure 6:**
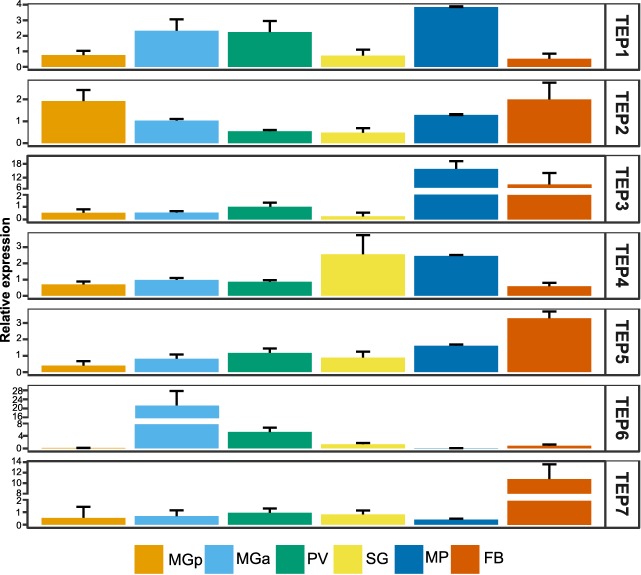
Tissue‐related thioester‐containing protein (TEP) expression in non‐infected *Glossina morsitans* flies. The relative expression of TEP transcripts in tissues dissected from non‐infected flies was determined by reverse transcriptase quantitative‐PCR in relation to ribosomal protein 49 (GMOY001799) and GMOY006676 as the reference genes. MGp, midgut posterior region; MGa, midgut anterior region; PV, proventriculus; SG, salivary glands; MP, mouthparts; FB, fat body. Values are means with SD, *n* = 5. [Colour figure can be viewed at http://wileyonlinelibrary.com]

To examine transcriptional profiles of tsetse fly TEPs we performed tissue‐specific reverse transcriptase quantitative‐PCR (RT‐qPCR) using cDNAs prepared from posterior midgut, anterior midgut, proventriculus, salivary glands, mouthparts and fat body tissues from adult non‐infected *G. m. morsitans* flies (Fig. 6). In all analysed tissues different *TEP* transcripts were detected. Of note, some *TEP* genes were abundantly expressed in specific tissues: (1) *TEP3*: mouthparts and to a lesser extent the fat body; (2) *TEP6*: mainly in the anterior midgut and to a lesser extent in the proventriculus; and (3) *TEP7*: mainly in the fat body. Interestingly, several TEPs were strongly expressed in the tsetse fly mouthparts.

### TEP expression in trypanosome‐infected tsetse flies

To examine the transcriptional profiles of the TEP genes in trypanosome‐infected tsetse flies, three different infection experiments were performed: (1) *G. m. morsitans* infected with *T. brucei*; (2) *G. m. morsitans* infected with *T. congolense* and (3) *G. p. gambiensis* infected with *T. brucei. T. brucei* and *T. congolense* have a similar life cycle in the tsetse fly midgut and proventriculus but the development of the final metacyclic infective stage occurs in a different tissue: *T. brucei* in the salivary glands, whereas *T. congolense* has its final development in the mouth parts of the fly. Therefore, the TEP expression analysis was performed in these tissues as they are relevant for the different parasite stages. Additionally, the fat body was also included in the analysis as it is a systemic immune response organ (although not directly infected with trypanosomes).

### TEP expression in *T. brucei*‐infected *G. m. morsitans* flies

**Figure 7:**
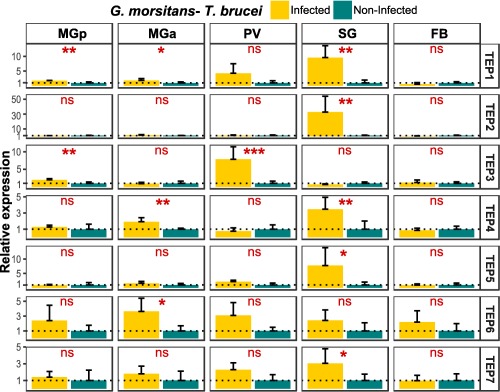
Tissue‐related thioester‐containing protein (TEP) expression in *Trypanosoma brucei*‐infected *Glossina morsitans* flies. The relative expression of TEP transcripts in tissues dissected from *T. brucei*‐infected flies and non‐infected flies was determined by reverse transcriptase quantitative‐PCR in relation to *ribosomal protein 49* (GMOY001799) and GMOY006676 as the reference genes (Supporting Information Table S3). MGp, midgut posterior region; MGa, midgut anterior region; PV, proventriculus; SG, salivary glands; FB, fat body. Values are means with SD, *n* = 5; unpaired *t*‐test, two‐tailed; ns, not significant; *, *P* <0.01 to 0.05; **, *P* < 0.001 to 0.01; *** *P* < 0.0001 to 0.001. Expression level for the samples obtained from non‐infected flies is set to 1.0, symbolized by the dotted line. [Colour figure can be viewed at http://wileyonlinelibrary.com]

To examine the transcriptional profiles of the seven *Gmm*_*TEP*s in *T. brucei*‐infected flies, we performed tissue‐specific RT‐qPCR using cDNAs prepared from salivary gland, proventriculus, anterior midgut, posterior midgut and fat body tissues, from flies harbouring a mature *T. brucei* infection vs. non‐infected, age‐matched flies (Fig. 7). The differential expression profiles were, in some cases, tissue specific. In the *T. brucei*‐infected posterior midgut, *Gmm*_*TEP1* (1.8‐fold change) and *Gmm*_*TEP3* (twofold change) showed significantly increased expression. *Gmm_TEP6* was significantly up‐regulated in the *T. brucei*‐infected anterior midgut, showing a more than fourfold increase. *Gmm_TEP1* (1.8‐fold change) and *Gmm_TEP4* (1.9‐fold change) were also found to be up‐regulated. *Gmm_TEP3* was highly expressed in the *T. brucei*‐infected proventriculus, with a more than sixfold up‐regulation. Five *TEP*s were significantly up‐regulated in *T. brucei*‐infected salivary glands. *Gmm_TEP2* was highly and specifically expressed in this tissue, with a more than 32‐fold change increase and the other four were *Gmm_TEP1* (9.6‐fold change), *Gmm_TEP4* (3.8‐fold change), *Gmm_TEP5* (7.7‐fold change) and *Gmm_TEP7* (threefold change). None of the analysed TEP genes showed differential expression in the fat body (Fig. 7).

### TEP expression in *T. congolense*‐infected *G. m. morsitans* flies

**Figure 8:**
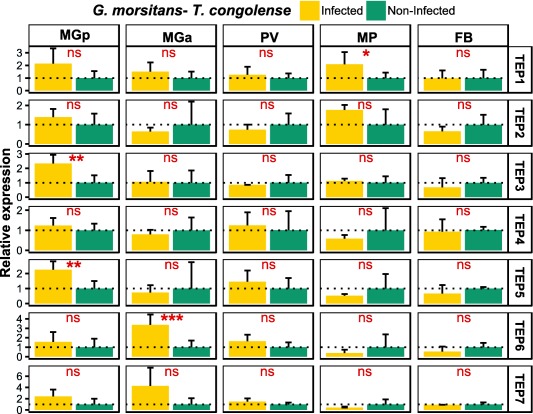
Tissue‐related thioester‐containing protein (TEP) expression in *Trypanosoma congolense*‐infected *Glossina morsitans* flies. The relative expression of TEP mRNAs in tissues dissected from *T. congolense*‐infected flies and non‐infected flies was determined by reverse transcriptase quantitative‐PCR in relation to *ribosomal protein 49* (GMOY001799) and GMOY006676 as the reference genes. MGp, midgut posterior region; MGa, midgut anterior region; PV, proventriculus; MP, mouthparts; FB, fat body. Values are means with SD, *n* = 5; unpaired *t*‐test, two‐tailed; ns, not significant; *, *P* < 0.01 to 0.05; **, *P* < 0.001 to 0.01; ***, *P* < 0.0001 to 0.001. Expression level for samples obtained from non‐infected flies is set to 1.0, symbolized by the dotted line. [Colour figure can be viewed at http://wileyonlinelibrary.com]

To investigate whether the observed differential *TEP* expression in *G. m. morsitans* flies was a general response to trypanosome infection or specific to the *T. brucei* parasite, a similar TEP expression analysis was performed in *T. congolense*‐infected and age‐matched non‐infected flies (Fig. 8). In the posterior midgut, two genes showed an increase in expression in the presence of the parasite, *Gmm_TEP3* (2.3‐fold change) and *Gmm_TEP5* (2.2‐fold change). In the anterior midgut only *Gmm_TEP6* was significantly up‐regulated in the presence of *T. congolense*, displaying a more than fourfold increase. Additionally, *Gmm_TEP1* presented an up‐regulation of more than twofold in the mouthparts. No differential expression was noted in the proventriculus, salivary glands and fat body in *T. congolense*‐infected flies.

### TEP expression in *T. brucei*‐infected *G. p. gambiensis* flies

**Figure 9:**
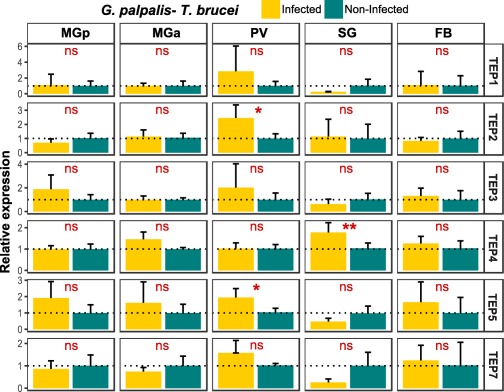
Tissue‐related thioester‐containing protein (TEP) expression in *Trypanosoma brucei*‐infected *Glossina palpalis gambiensis* flies. The relative expression of TEP mRNAs in tissues dissected from *T. brucei*‐infected flies and non‐infected flies was determined by reverse transcriptase quantitative‐PCR in relation to *ribosomal protein 49* (GPPI037276) and *ribosomal protein L13a* (GPPI017876) the reference genes (Supporting Information Table S3). MGp, midgut posterior region; MGa, midgut anterior region; PV, proventriculus; SG, salivary glands; FB, fat body. Values are means with SD, *n* = 5; unpaired *t*‐test, two‐tailed; ns, not significant; *, *P* < 0.01 to 0.05; **, *P* < 0.001 to 0.0. Expression level for samples obtained from non‐infected flies is set to 1.0, symbolized by the dotted line. [Colour figure can be viewed at http://wileyonlinelibrary.com]

TEP gene expression was also determined in another tsetse fly vector, *G. p. gambiensis*, which was infected with the *T. brucei* parasite and compared with age‐matched non‐infected flies (Fig. 9). As described above, the genome of *G*. *p*. *gambiensis* harbours six TEP genes (Fig. 3), there is no TEP6 orthologue. Three TEP genes were found to be significantly up‐regulated following *T. brucei* infection: *Gpg*_*TEP2* (2.5‐fold change) and *Gpg*_*TEP5* (1.9‐fold change) in the proventriculus and *Gpg*_*TEP4* (1.8‐fold change) in the salivary glands. No differential expression was observed in the posterior and anterior midgut or fat body of *T. brucei*‐infected flies.

## Discussion

In the present study, we report for the first time a detailed genomic characterization of the TEP family from six tsetse fly species and a possible role of this protein family in the interaction with the trypanosome parasite population. Overall, depending on the species, the genome of the tsetse fly contains six to eight genes encoding for TEP proteins belonging to two specific TEP subfamilies ie insect TEP and MCR proteins (Fig. 4A), a similar repertoire as observed in *D. melanogaster* (Lagueux *et al*., 2000). The majority of *Glossina* TEP genes are located on the same scaffold and analysis of the gene structure (Fig. 3) suggests an expansion through segmental duplication (Hurles, 2004). A similar feature has been observed in the *An*. *gambiae* mosquito genome, where the 15 TEP genes are organized in four clusters and one isolated locus (Christophides *et al*., 2002). Furthermore, phylogenetic analysis revealed that this gene duplication gave rise to an expansion of specific tsetse TEPs that are not found in any other dipteran (Fig. 4A). Many similar genus‐specific gene duplications can be found in the Brachycera suborder. Gene family duplication is one of the shaping forces in the evolution of an organism but understanding the factors maintaining paralogues in a genome is a difficult task (Innan & Kondrashov, 2010). One feature that may drive paralogue maintenance is host–pathogen interaction. Indeed, it has been observed that immune genes are experiencing rapid evolution in many organisms, armouring hosts with defence mechanisms to detect and fight pathogens. Such expansion in the TEP protein family has been documented in the tsetse fly and stable fly (this paper, Fig. 4A), mosquitoes (Little & Cobbe, 2005; Obbard *et al*., 2008), fruit fly (Sackton *et al*., 2007) and in the house fly (Sackton *et al*., 2017). Interestingly, these species are either blood feeders (like mosquitoes, tsetse and stable fly) or various pests such as fruit flies and house fly, species with a feeding habit that exposes them to a rather pathogen‐unpredictable environment. In contrast, insects feeding on plants show no such TEP gene expansion in their genome; eg various species of bees (*Apis*, *Bombus*) have only three *TEP* genes (Bou Aoun *et al*., 2011; Palmer & Jiggins, 2015), the silk worm (*Bombyx mori*) has four genes (Zhao *et al*., 2012) and tobacco hornworm (*Manduca sexta*) two genes (Gunaratna & Jiang, 2013).

In arthropods, blood‐feeding behaviour evolved independently more than 20 times and in insects at least five times at the order level (Mans, 2011). Consequently, in these organisms specific mechanisms and unique protein repertoires have evolved to acquire and process efficiently the bloodmeal, as well as the microbial challenge associated with it (ie ingestion of microbial contaminants during blood feeding on the host skin; Ribeiro & Arca, 2009). Expansion of the TEP protein family observed in various blood‐feeding insects could probably be an adaptation to their respective life styles. As an exception, sand fly genomes (*Lutzomyia longipalpis* and *Phlebotomus papatasi*) encode only three TEPs: one MCR gene and two iTEP genes (Fig. 4A).

The TEP genomic arsenal in the tsetse species investigated here showed variations between subgenera (groups). Both Palpalis group (*G. p. gambiensis*, *G. fuscipes*) and Fusca group flies (*G. brevipalpis*) lack the *TEP6* gene whereas the latter also showed two additional TEPs (*TEP8* and *TEP9*) that were not found in the other genomes. Whether TEP expansion in the tsetse groups is somehow associated with their biological differences regarding feeding preference or ability to transmit the trypanosome parasites remains to be determined. In our phylogenetic analysis (Fig. 4A), *Gb_TEP8* and *Gb_TEP9* formed a separate clade, indicating that they emerged from a *Gb_TEP5* duplication. It would be interesting to see if this expansion is Fusca specific or restricted only to *G. brevipalpis*.

The main characteristic of the TEP protein family is the presence of a thioester covalent bond between the sulphydryl group of cysteine and the carbonyl group of glutamine (Tack *et al*., 1980; Law & Dodds, 1997). This thioester site (CGEQ) is a functionally essential site that allows the formation of a covalent bond to microbial surfaces and variations in this site have been linked with the inability of the protein to bind to microbes. In the tsetse TEP repertoire, five proteins (TEP1, TEP5, TEP6, TEP7 and TEP8) have an intact thioester motif, whereas TEP2 and TEP3 show variations that could hamper their microbe‐binding functionality. Tsetse TEP2 sequences lack the cysteine in the thioester site and are hence presumably not able to bind to microbes. These proteins are part of the ‘ancestral iTEP’ group (Fig. 4A), clustering together with fruit fly *D. melanogaster*_TEP3 where the intact motif is still present. The presence of the cysteine in the presumed ancestral tsetse species, *G. brevipalpis*, indicates that mutations took place after the separation of the Morsitans group, where two nucleotides resulted in its replacement with serine and later on in the Palpalis group a new mutation resulted in its replacement with asparagine. In addition, in the Fusca group (*G. brevipalpis*) the glutamine residue is replaced by a histidine. Overall, these results suggest that none of the tsetse TEP2 proteins can form a thioester bond and therefore might not have a microbe‐binding capacity. *An. gambiae_*TEP1 has been shown to be a key molecule in the immune response against malaria parasites. This molecule is capable of binding *Plasmodium* ookinetes in the midgut epithelium basal lamina and targeting them for lysis (Blandin *et al*., 2004). Interestingly, the presence of the thioester motif is not always a prerequisite for pathogen binding. *D. melanogaster*_MCR was shown to specifically bind to the *Candida albicans* surface, and to subsequent promote its phagocytosis (Stroschein‐Stevenson *et al*., 2006). Moreover, not all TEPs directly interact with the pathogen surface. *Ae. aegypti_*MCR was reported to control dengue virus infection in an indirect manner, by induction of antimicrobial peptides after its recruitment of a scavenger receptor‐C (Xiao *et al*., 2014). However, whether the TEP‐based complement‐like system plays a role in tsetse fly defence against trypanosomes remains to be determined.

Overlapping phylogenetic data with the results of differential gene expression studies is one of the first steps in understanding the possible function of the corresponding proteins. Therefore, we first investigated the expression profiles of the different *TEP* genes in various tissues of non‐infected adult *G. m. morsitans* flies. All of the *TEP* genes were found to be expressed (at variable levels) in different parts of the alimentary tract and systemically in the fat body. Remarkably, strong expression of some TEPs was found to be tissue‐specific ie *Gmm_TEP3* (mouthparts, fat body), *Gmm_TEP6* (anterior midgut) and *Gmm_TEP7* (fat body) (Fig. 6). In *D. melanogaster* adult flies, the tissue‐specific TEP expression pattern was found to be consistent with a role in innate immunity as TEP genes were expressed in haemocytes, in the fat body and in some barrier epithelia (Bou Aoun *et al*., 2011). The elevated expression of TEP genes in the alimentary tract of tsetse fly could also be suggestive of an innate immune function with an involvement in the inactivation of a variety of microorganisms that are ingested when feeding on a host, followed by neutralization and hindering their proliferation/invasion to other tissues.

In addition, our data suggest a differential expression of TEPs in different parts of the tsetse alimentary tract in response to the trypanosome parasite during its complex developmental cycle in the fly (Rotureau & Van Den Abbeele, 2013). The significantly increased expression of *G. m. morsitans* TEPs upon *T. brucei* or *T. congolense* infection in the different tissues may indicate their involvement in the control of the parasite in the different tsetse micro‐environments. Remarkably, a trypanosome‐specific TEP response was observed, indicating the existence of a differential parasite‐recognition mechanism.

In the posterior midgut, three TEP genes were observed to be up‐regulated. *Gmm*_*TEP3* had increased expression both in response to *T. brucei* and *T. congolense* whereas two other genes showed a trypanosome‐specific response: *Gmm*_*TEP1* to *T. brucei* and *Gmm*_*TEP5* to *T. congolense*. From the posterior midgut, it has been suggested that the established procyclic trypanosomes cross the peritrophic matrix, and migrate to the anterior part of the midgut. Here, we found significant up‐regulation of *Gmm_TEP6* in response to both parasites, whereas *Gmm_TEP4* was up‐regulated only in response to *T. brucei*. Differential expression of TEP genes in *T. brucei*‐infected midgut was also observed in a macroarray analysis (Lehane *et al*., 2003), where two genes corresponding to *Gmm_TEP6* and *Gmm_TEP3* were found to be up‐ and down‐regulated, respectively.

From the anterior midgut trypanosomes invade the proventriculus (cardia), where several differentiation steps take place. Beside its role in the synthesis of the peritrophic matrix components (Lehane, 1997), this tissue can regulate the expression of some immune effectors like antimicrobial peptides and reactive oxygen/nitrogen species (Hao *et al*., 2003). Here, *Gmm_TEP3* was found to be highly up‐regulated in response to *T. brucei* but not to *T. congolense*. *T. congolense* parasites migrate to the mouthparts (cibarium and proboscis) and attach to the chitinous lining where they develop into an infective metacyclic form. Currently, knowledge on immune‐related tsetse–trypanosome interactions in the cibarium and proboscis is lacking. Therefore, our finding of *Gmm_TEP1* up‐regulation in response to *T. congolense* in the mouthparts is the first of its kind. In contrast to *T. congolense*, *T. brucei* parasites do not establish in the tsetse mouthparts but migrate to the salivary glands, where they attach to the gland epithelium and complete their development into the infective metacyclic form. Consistent with our previously reported RNA‐seq results (Matetovici *et al*., 2016), there is strong up‐regulation of different TEPs (especially TEP2) in the *T. brucei*‐infected salivary gland. None of these TEP genes were found to be differentially expressed in the salivary glands from *T. congolense*‐infected flies (data not shown), suggesting that the TEP response is locally induced by the parasite in the gland. In both *T. brucei*‐ and *T. congolense*‐infected flies none of the TEP genes were up‐regulated in the fat body. A similar result was observed in the *D. melanogaster* abdominal fat body where no expression of TEP genes was detected before or after systemic immune challenge (Bou Aoun *et al*., 2011).

Of note is the significant up‐regulation of *Gmm_TEP4* (MCR), especially in the salivary glands, of *T. brucei*‐infected flies. In *D. melanogaster*, the MCR has been shown to have multiple roles in innate immunity (Stroschein‐Stevenson *et al*., 2006; Shokal *et al*., 2017) and in the formation and maintenance of the septate junctions (Batz *et al*., 2014; Hall *et al*., 2014). In *T. brucei*‐infected salivary glands, the parasites tightly attach with their flagellum to the epithelial lining (Vickerman *et al*., 1988), thereby possibly damaging the septate junctions. Increased expression of *Gmm_TEP4*, together with the other eight genes encoding for septate junction proteins (Matetovici *et al*., 2016), would consequently be needed to repair and maintain the structural integrity of this tissue. *Gmm*_*TEP4* up‐regulation in salivary glands was not observed in *T. congolense*‐infected flies (data not shown), again demonstrating the specificity of this parasite‐related response.

The Morsitans group flies are known as good vectors of animal pathogenic trypanosomes (especially *T. congolense* and *T. brucei brucei*) and of the human‐pathogenic *T. brucei rhodesiense*, in contrast to the Palpalis group flies, which are more refractory to trypanosome infection despite being the main vectors of the human *T. brucei gambiense* parasite. Our study revealed that the TEP expression profile in response to a trypanosome infection (*T. brucei brucei*) is strongly dissimilar in the two tsetse fly species, with only few genes up‐regulated in *G. p. gambiensis* flies in sharp contrast to *G. m. morsitans* flies. Significant differences between these two species in the midgut immune‐related response following trypanosome infection have been previously reported (Hamidou Soumana *et al*., 2014). Similar to our results, no TEP genes were found to be differentially expressed in a recent comparative transcriptome analysis of *T. b. gambiense‐*infected midgut (Hamidou Soumana *et al*., 2015). Altogether, these results suggest differences in the innate immune responses of these two vectors to trypanosomes and strengthen the need for more comparative studies between the tsetse species.

The analysis of the tsetse TEP sequences revealed interesting information about their structure, evolutionary relationships and expression profiles under both normal and trypanosome infection conditions. We demonstrated the occurrence of a genomic expansion of specific tsetse TEPs that are not found in other dipterans. Moreover, we found general expression of all TEP genes in the alimentary tract, mouthparts and salivary glands, and a trypanosome‐specific and tissue‐related TEP response in the tsetse fly. These findings are suggestive for the involvement of the TEP family in tsetse innate immunity, with a possible role in controlling the parasite density in the infected tissues and/or acting in tissue damage repair.

## Experimental procedures

### Sequence retrieval, identification and annotation of tsetse fly TEPs

Genome sequences of six tsetse species, *G. m. morsitans*, *G. pallidipes*, *G. austeni*, *G. fuscipes*, *G. p. gambiensis* and *G. brevipalpis*, were obtained from the VectorBase database (https://www.vectorbase.org/; genome version is described in Supporting Information Table S1).

Exon–intron structures were predicted using GenScan software (http://genes.mit.edu/GENSCAN.html). The signal peptides of tsetse fly TEP amino acid sequences were predicted via the SignalP program (http://www.cbs.dtu.dk/services/SignalP/) and the protein domains by the smart program (http://smart.embl-heidelberg.de/).

### Alignments and phylogenetic analysis

Sequences of the TEPs from *D. melanogaster* were retrieved from FlyBase (http://flybase.org/). Sequences belonging to Muscidae, Culicidae, Psychodidae, Cimicidae, Reduviidae, Sarcoptidae and Ixodidae species were retrieved from VectorBase by blastp searches using the annotated *D. melanogaster* sequences. The Tephritidae sequences were retrieved from the NCBI protein database after they were identified by NCBI blastp searches (https://blast.ncbi.nlm.nih.gov/Blast.cgi) using annotated *D. melanogaster* sequences. The obtained protein sequences (Supporting Information Table S2) were used to create a multiple alignment with the muscle algorithm (http://www.ebi.ac.uk/Tools/msa/muscle/). The phylogenetic tree was constructed using the ML method implemented in PhyML (Guindon *et al*., 2010), with the best‐fitting evolutionary model and 100 bootstrap replications. The trees were displayed and annotated with Interactive tree of life (iTOL) v. 3 (Letunic & Bork, 2016).

### Tsetse fly infection and tissue collection

Male *G. morsitans morsitans* from the colony at the Institute of Tropical Medicine (Antwerp, Belgium) were used in all experiments (Elsen *et al*., 1993). The *G. palpalis gambiensis* flies emerged from pupae obtained from the tsetse fly colony of the Institute of Zoology of the Slovak Academy of Sciences (Bratislava). All experimental flies were maintained at 26 ± 0.5°C and 65 ± 5% relative humidity.

The *T. brucei brucei* AnTAR1 (derived from EATRO 1125; Le Ray *et al*., 1977) and *T. congolense* MSOROM7 (Tihon *et al*., 2017) strains were used for the tsetse fly infection experiments. Freshly emerged flies were fed 24–48 h after their eclosion with a trypanosome‐infected bloodmeal supplemented with 10 mM reduced L‐glutathione to enhance midgut infection rates in the tsetse fly (MacLeod *et al*., 2007). For this, parasitized blood was harvested with heparin from cyclophosphamide‐immune suppressed mice (Endoxan^®^, Baxter, Germany) at 6–7 days postinfection and mixed with defibrinated horse blood (E&O Laboratories Limited, Scotland, UK) to obtain around 2 × 10^6^ bloodstream form trypanosomes/ml in the initial bloodmeal. Afterwards, the flies were maintained for 4 weeks and fed every 2–3 days on non‐infected defibrinated horse blood using an artificial membrane feeding system. Three different tsetse fly‐trypanosome infection experiments were performed: (1) *G. m. morsitans*–*T. brucei*; (2) *G. m. morsitans*–*T. congolense* and (3) *G. p. gambiensis*–*T. brucei*.

Twenty‐eight days after the infective bloodmeal, *T. brucei* salivary gland infected flies were selected by induced probing on prewarmed (37 °C) glass slides, which were microscopically examined for the presence of metacyclic trypanosomes (modification of the method of Burtt, 1946). Immediately after screening the flies were fed and maintained for another 72 h before dissection. To select for the *T. congolense‐*infected flies, individuals were microscopically examined during dissection and only infected tissues were collected. For each of the three fly infection groups, a control group of age‐matched and non‐infected tsetse flies was maintained on the same feeding regime. From all these experimental groups the following tissues were collected by dissection: salivary glands (pool of 20); proventriculus (pool of 10); anterior midgut (pool of three); posterior midgut (pool of three) and fat body (pool of three).

### RNA isolation and TEP tissue expression profiling by RT‐qPCR

Total RNA was isolated from the pooled tissues using TRIzol® reagent (Life Technologies, Rockville, MD, USA) according to the manufacturer's instructions. Samples were DNAse I treated (Ambion, Life Technologies, Rockville, USA) and first‐strand cDNA was reverse transcribed using oligo(dT)15 primer and Transcriptor Reverse Transcriptase (Roche, Indianapolis, IN, USA), following the manufacturer's instructions. RT‐qPCR reactions of 20 µl were performed with a SensiMIX^TM^ SYBR® No‐ROX kit (Bioline, London, UK) and 0.5 µM of each primer (Supporting Information Table S3). They were run on a Light Cycler 480 system (Roche) with the following thermal cycling conditions: 10 min at 95 °C (polymerase activation) followed by 40 cycles of 10 s/95 °C (denaturation), 10 s/60 °C (annealing) and 30 s/72 °C (elongation). For each tissue five biological replicates were used. Data were analysed using BioGazelle qbase^+^ version 1.5 software (Biogazelle NV, Belgium) in order to evaluate reference gene stability and to obtain normalized values for the tested genes in the different tissue samples.

### *G. m. morsitans* TEP6 cloning

The missing *Gmm_TEP6* sequence was obtained by PCR using the following primer set: pET22b_Tep6_Fw 5'‐agtggtggtggtggtggtgctcgagTGTGGAACTAGATCACAGGCTTCATCTGGC‐3' and pET22b_TEP6_Rev 5'‐ctttaagaaggagatatacatatgAGCGTGTCCTTCATGATACGTCCAACTGTAG‐3' (vector sequence is in lowercase and gene‐specific sequence in uppercase). The PCR reaction was set up as follows: 1× Phusion Master Mix (New England Biolabs, Beverly, MA, USA), 100 ng cDNA from *T. brucei*‐infected midgut (obtained as described above) and 0.5 μM of each primer. The PCR cycling conditions were as follows: 1 min at 98 °C for initial denaturation, followed by 30 cycles of 15 s at 98 °C (denaturation), 30 s at 72 °C (annealing) and 60 s at 72 °C (elongation), and another 10 min at 72 °C (final elongation). The amplification product was cloned into linearized (*Xho*I and *Nde*I restriction enzymes) pET22b vector (Novagen, Madison, WI, USA) using a NEBuilder HiFi DNA Assembly Cloning kit (New England Biolabs) and transformed into NEB^®^ 5‐alpha Competent *Escherichia coli* cells. Four positive clones were Sanger sequenced using T7 promoter/terminator primers. After sequencing a new internal primer (Tep6_Fw: AACCGCTTATGTGGCTCGTT) was designed and the plasmid was resequenced, covering in this way the entire gap.

## Supporting information

Additional Supporting Information may be found in the online version of this article at the publisher's website:

**Table S1.** *Glossina* spp. thioester‐containing protein genes information (sheet 1) and tsetse species genome data (sheet 2).

**Table S2.** Species used in the phylogenetic tree.

**Table S3.** Reverse transcriptase quantitative‐PCR primers used for thioester‐containing protein (TEP) expression analysis. *Glossina morsitans* TEP genes (sheet 1); *G. m. morsitans–Trypanosoma brucei* reference genes (sheet 2); *G. p. gambiensis* TEP genes (sheet 3) and *G. p. gambiensis–T. brucei* reference genes (sheet 4).

**Figure S1.** Multiple sequence alignment of *G. m. morsitans* insect thioester‐containing proteins (iTEPs). The conserved functional domains including four proteinase‐binding alpha‐2‐macroglobulin (α2M) domains (A_2_M_N, A_2_M_N2, A_2_M and A_2_M_comp), one A_2_M receptor binding domain (A_2_M_receptor) and a thioester domain (Thioester) are marked on the top of the alignment with a blue line. The signal peptide sequence at the N‐terminal is in italics and the putative thioester motif (GCGEQ) is highlighted in black. The thioester associated histidine (His) is indicated by an arrow at position 1991. Six cysteines located at 1392–1530 in the C‐terminal are highlighted in yellow.

**Figure S2.** *G. m. morsitans* thioester‐containing protein sequences.
