# Supplementary material for: Thioester‐containing proteins in the tsetse fly (Glossina) and their response to trypanosome infection
Source: Insect Mol Biol. 2018 Mar 12;27(3):414–28. doi: 10.1111/imb.12382 (PMC5969219; doi:10.1111/imb.12382)
Supplement: Supplementary file 4 — Figure S1. Multiple sequence alignment of G. m. morsitans insect thioester‐containing proteins (iTEPs). The conserved functional domains including four proteinase‐binding alpha‐2‐macroglobulin (α2M) domains (A2M_N, A2M_N2, A2M and A2M_comp), one A2M receptor binding domain (A2M_receptor) and a thioester domain (Thioester) are marked on the top of the alignment with a blue line. The signal peptide sequence at the N‐terminal is in italics and the putative thioester motif (GCGEQ) is highlighted in black. The thioester associated histidine (His) is indicated by an arrow at position 1991. Six cysteines located at 1392–1530 in the C‐terminal are highlighted in yellow. [file IMB-27-414-s004.pdf]

```
                  10        20        30        40        50        60        70        80        90       100       110       120
         ....|....|....|....|....|....|....|....|....|....|....|....|....|....|....|....|....|....|....|....|....|....|....|....|
Gmm_TEP2 MFYTKYSSLTLIIILVHFCAGQGL---------YSIIAPNTLRPNSQYHVAVSIHKASEPVKVKIGILGSTYSESKTIEVRPFSTELIEFEIPALKNDRYRLVAEGLTGIN-FANETQLN
Gmm_TEP3 ---MKLSFIEDLEILI-DTSTAGLYITGSFFLKYSVIGPGTIHSDGKYTVAVAVHHVAEPCQIQVGLTGPSYNDSKIVELSGFEVKNVDFDLPVLERGDYNLTAEGLNCMKMFKNSTKLN
Gmm_TEP1 -------------------MSEPI---------YTIVAPDTIRSHQKFPVSVTLHDAQTTVTLDISVTGPSYNQSKIVNLSSMENKQIDFDVPALCDGSYQLTSKGIEGLQ-IEKSTALY
Gmm_TEP5 MWQNRM--YILLHMLC-VVNANGI---------YSIVAPGSIYSNRKYSVSVTLHDAGQAVTFNIGISGPSYNHSKSIELSPKENKRIDFNVPELKKGIYQLVSKGIGGLY-FENTTYLS
Gmm_TEP6 MFQNICLVYCILHLLW-LVRGSGI---------YSIVAPKTLQSNHKYSVSVTLHDAKQPVTLNIGITGPSYNHSETVNLTAIETTQIDFVLPALDGGPYRLITKGIEGLD-FENATELH
Gmm_TEP7 MFQNFYLVYCILHMMW-LARGNGI---------YSIVAPKNLQSNHKYSVSVTLHDAKQPVRFNIGITGPSYNHSETVNLTPIETTQIDFVLPELDGGPYLLISKGVEGLD-FENATELH

                 130 A2M_N   140       150       160       170       180       190       200       210       220       230       240
         ....|....|....|....|....|....|....|....|....|....|....|....|....|....|....|....|....|....|....|....|....|....|....|....|
Gmm_TEP2 FDHKQHTVLVQTDKAIYKPTDLVQFRILIMDANLKPARN-YPNTHITIRDGGDNIIRSDRDVHIISGVYANDLLLADYPKFGEWSIEVQVGDEIYK-RSFEVVEYILPKFVVDITTEKHV
Gmm_TEP3 YSKFHTNVRVQTDKGLYKPGDVINYRVIFLDKNLRPDKP-IKEAKIYVEDGKRNRIKEIKDFNVVQGVYTGKFQISEYPVTGGWRLGVSNGGRYDHMVYFDVDKYVLPKYVVKVESTERV
Gmm_TEP1 MDTNQPNIYIQTDKAVYKPGDLVQYRILILDENIRPVKL-ERPLRVAIKDAANNYIKEFKVSHLTKGVYSGRFQLTEQPILGQWTIEVDLAKDAQQEKNFKVIKYVLPRFSVDIETVKDL
Gmm_TEP5 VEYTRPNLYIQTDKAMYKPGDLVQYRILILDENFRPLKS-DRSLGVAIKDAANNLVKDIKNAENYKGVFSDKLQLTEQPVLGLWIIEVSLNDHIEKTKEFEVAKYVLPKFSVDIDAVTDL
Gmm_TEP6 VTQSTSNVYIQTDKAMYKPGDLVQYRILVLDENLRPLKS-DRPLGVAIKDAANNLIKDIKNVQLIKGVFSDKLQLTEQPVLGLWIIEVSLSDHLEKTKQFEVAKYVLPKFSVDIDAVTDL
Gmm_TEP7 VTQSTSNVYMQTDKAMYKPGDLVQYRILVLDETLRPTKLKEASLKVTIKDAQNNLVRDAKNVKLIKGVFSDKLQLTEQPVLGVWKIEVSLNDHEDKTKTFEVAKYVLPRFSVDIDTVTDL

                 250       260       270       280       290       300       310       320       330       340       350       360
         ....|....|....|....|....|....|....|....|....|....|....|....|....|....|....|....|....|....|....|....|....|....|....|....|
Gmm_TEP2 IYKDNKISASIKAYYMFGKPIVGEATLSIYPTFFGSL---------QPFVNDLITRKVMPIDGSAYFEFDIRNELKL--KEDYEREYLLDALVEERSTGSVQNFSTIITVHLDPYKVEAI
Gmm_TEP3 SVKDGDMQVIVRANYTYGKPLNGKVTLLVNLNVNRYYYRGDSETEETPKNPPTIIKTAPMIQGKSKIDLDVKEYEAFMDSKTSPSYLSIVATVEEEFTGVKINATSGSTVYPYRYSMNCI
Gmm_TEP1 AINDNSLKVAVCAKYTYGKPVKGKAIITI---------------------LYLNLEKIIDINGKEEVEFTLHKGLNW---NSLGENMTISAIVDEELTGNRQSNTIDIKLHSSQYVVKML
Gmm_TEP5 AITESSLTITVRAKYTYGKPVKGKATVHL---------------------SPVDLEKTIDINGKGHVEFDLKKDLNLIVSNRFVRELKVFAVVEEELTGNRQNTTLKINLHRSPYRIEVS
Gmm_TEP6 AITESSLKITVRAKYTYGKPVKGKATVRL---------------------SPINLEKSVDMNGKGHVEFDLRKDLNI-IREESVRELKAFAMVEEELTGNKQNATVKINLHHSPYIIEVF
Gmm_TEP7 AITERLLKVTVRAKYTYGKPVKGKATVRL---------------------SPVNLEKTIDVNGKGHVEFDLQKELKAAVKEGAVE---IFAAVQEDLTDSRQNNTIIVNFHSSQYIIKTP

                 370       380       390       400       410       420       430       440       450       460       470       480
         ....|....|....|....|....|....|....|....|....|....|....|....|....|....|....|....|....|....|....|....|....|....|....|....|
Gmm_TEP2 --KMPRYYIPGIPFDVTVRVTSNVGDRTKDLKSQL----TAYLTNVYGSSEIYNKT----------------VY--NLDEQSEVKMKFTVPA----GDRDEYHSVIVDYMGIITDI----
Gmm_TEP3 SYDTCSVFQADKEAEVEFQIVYVDGTHLNDTKSPVELIYTEVLNKYRVWYPDSDEENSKEADTEPVSENRTFHFRSHMNESSIAVFKVSLPDLRDYRKHAHFYKMELKYRDEQRELYSTY
Gmm_TEP1 --DSVIEFEINEPFVVKAAVEYLNGDPVRNAKDPI------FLKYYRGWGEPEESQ----------------LFESTLDDNGVGMFKVNLPN-------GGIYLGELRFMDKVEIL----
Gmm_TEP5 --DMMKEFEINQTIEVKVVIKYLNGNPVQDTKAPV------LLKFYNTRRADEDPE----------------IFKATLDEHGVVIFKISFQN-------DGFYWPELKFAEEIKHM----
Gmm_TEP6 --DVMTEHEVNQPFEVKVVIKHLNGKPVQDTKTPV------LLNYYYRWGEYKNAE----------------IFRANLDEHGVAIIKVNFQK-------DGFYWCELKFADEVKRL----
Gmm_TEP7 --NAVGEFEPNKPFEVKAVIQYLNGKPVRDAKTPA------LLHCYQGWGGNIKSE----------------IFKAKLDDHGEAIFAVKLPQ-------ASVFRCEIEFADKTVHL----

                 490       500       510 A2M_N_2 20       530       540       550       560       570       580       590       600
         ....|....|....|....|....|....|....|....|....|....|....|....|....|....|....|....|....|....|....|....|....|....|....|....|
Gmm_TEP2 ----GKVPSKYIGGK--NFIISKII-------TEKPVINQEIGVMVRCNEPIKYFIYQLVGRGDILLSRSVEVSDSTQ-YTFKFLATFAMMPRAKLLIYTVIN-GELVYDELDVELEESL
Gmm_TEP3 QYREPKNLDPLSAEE--NDKLKEFFQLEYKRYDDKIEINKESQFTVNSSQPLSYVVYNVVGRGNILKSDRIDLPDKPKFHNISLTPTEMWAPNFALYVYYVDEKGEYHYAEQRYYVQYRL
Gmm_TEP1 ----PCIMAKSHNTTLAQAEIREELTLVLN--TERPRIGDDASVTVKAPNMMTHLTYVIVGRGCILQTAYITLPEPANFYKINFKVTFEMIPRADVFVFYVDK-SDLKYQEISIDFELEF
Gmm_TEP5 ----PSISVRAASEK--NTKLVSQLTLELE--TIKPRLGEHVSIAVKAPDVMNHLIYAVVGHGTILQIANISLPRPQQFYKVTFRATFEMMPRANLFVYYVDE-SDLKFQEITVEFLPEF
Gmm_TEP6 ----PSVGVAAARKK--NTEL---LTLELE--TIRPQLGEYVSIAVKAPKVMTHLIYTVVGRGNILHKRNIPLPNPQNFYTISFKTTFEMIPKVHVFVYYVDE-GDLKFEEIGVKIQPEF
Gmm_TEP7 ----PPIHRKIDENN--FVKMTRELTLTLV--DKTPRVGEDVSIEVKASNPFSYLVYVIVGRGNILQMRNIPLPNPQNFYTITFKSTFEMIPNAHVFVYYVDN-GDLKFQEIGVKIQPEF

                 610       620       630       640       650       660       670       680       690       700       710       720
         ....|....|....|....|....|....|....|....|....|....|....|....|....|....|....|....|....|....|....|....|....|....|....|....|
Gmm_TEP2 LNNVRIEVPSTASPGQDIDISIAAKPYNYIGLMIVDQNAINLREGNDLSVKSLMRALNDYELSDVN--TPIST----PGKLSGVITLTNADFWPVQESFTTMENPAYHEDHKLTTIRKTD
Gmm_TEP3 QNQINITAPEQVKPGENVSLKIKTAPNSFVGLTAVDQSVLLLRSNNDLRPHEFDWVLSSYTTTTPHQGGYSDY----PGWSSGVVTLTNADY-FYN--WTKPEYLSTPLSAQLDSELNNR
Gmm_TEP1 QNSIKLTGPLQAKPGQEVSLDIETVSNSFVGLLGVDQRTLLLERGNDFERNTILNNLRHHNTNI----GFFPY----PGKMSGLAVQTNARF-PYE------------------------
Gmm_TEP5 ENKIEITGPIQATPGQEVSLEIKTDPNSYIGLLGVDQSMLLLKSGNDLEFAAILNDLRNRKPIDKH----------------------------YQNTRLKKGASHYEIEEEMSESVDDD
```

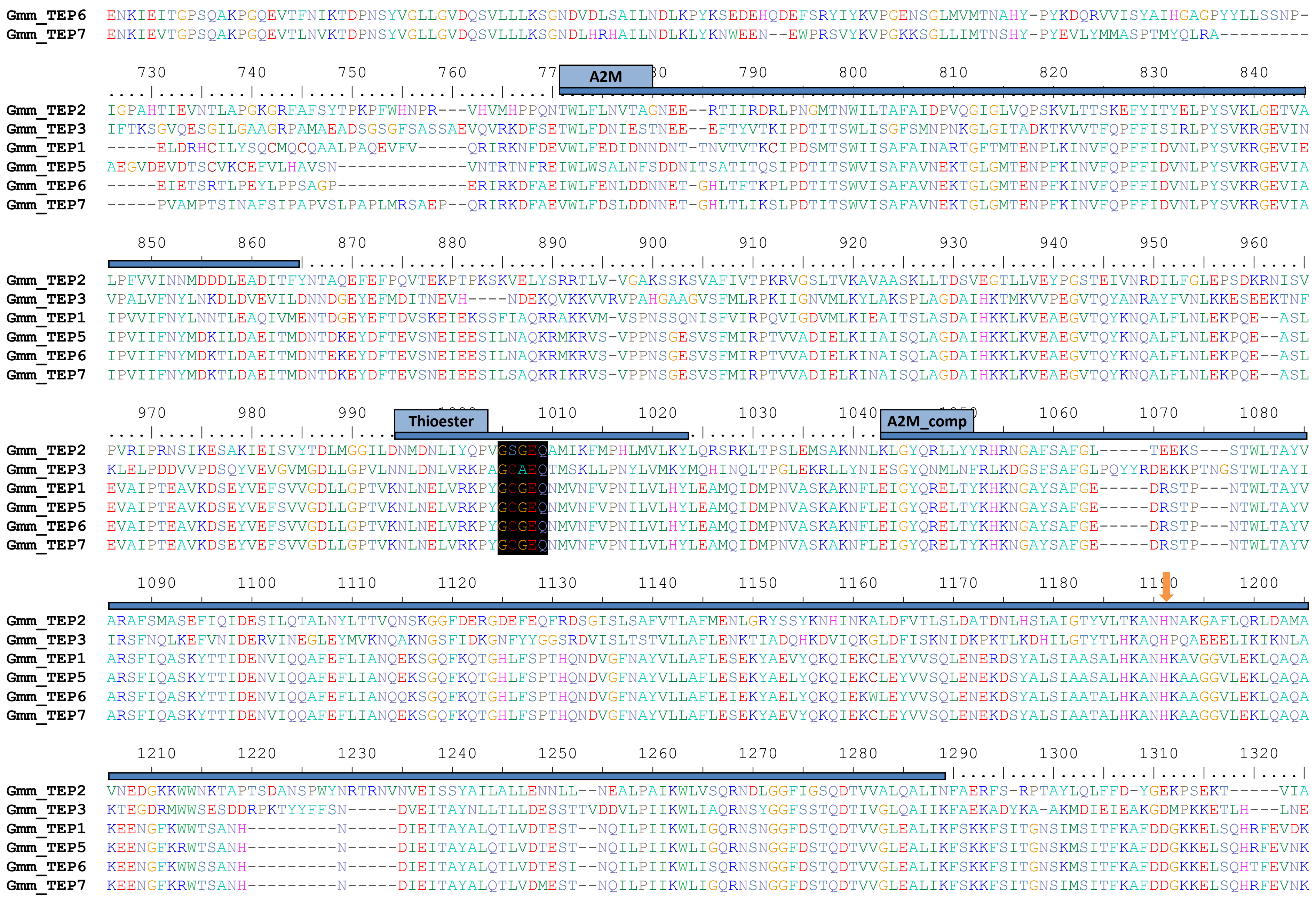

Gmm_TEP1
Gmm_TEP2
Gmm_TEP3
Gmm_TEP5
Gmm_TEP6
Gmm_TEP7
A2M
Thioester
A2M_comp

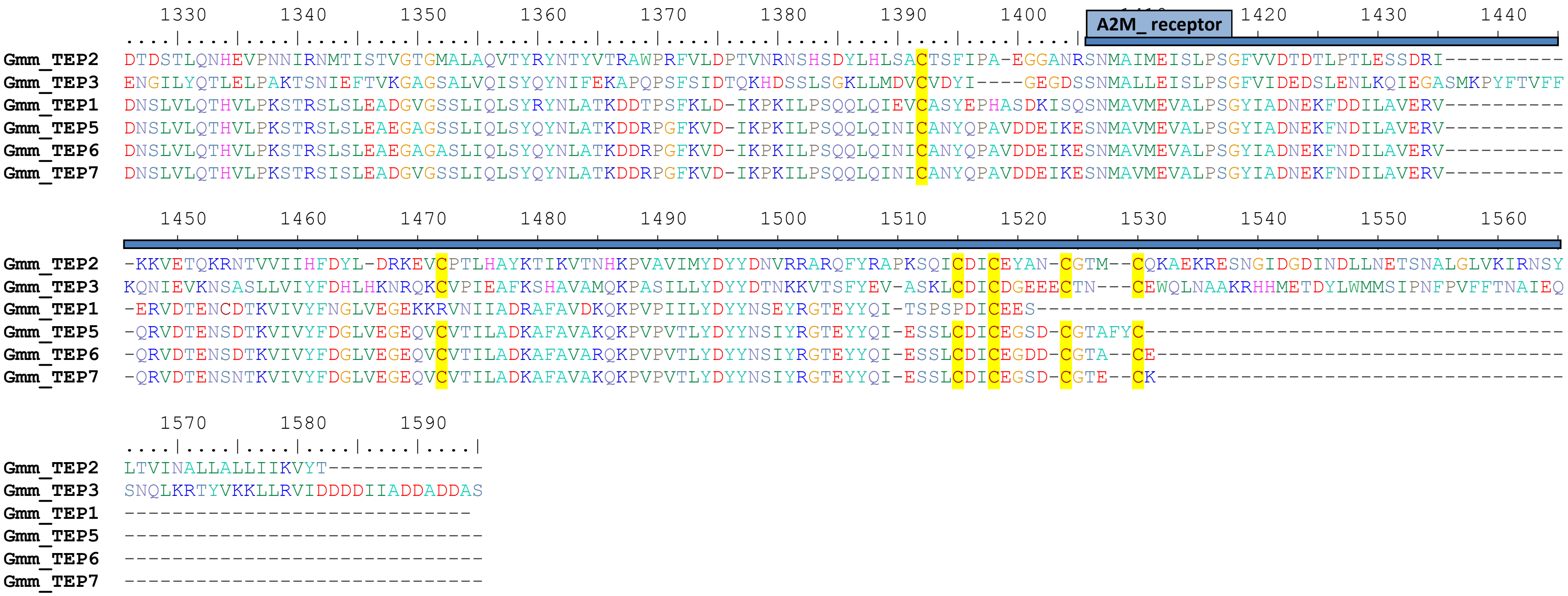

Figure_1_SuppInfo Multiple Sequences Alignment of *G. morsitans* insect thioester-containing proteins (iTEPs). The conserved functional domains including four proteinase-binding alpha-2-macroglobulin (a2M) domains (A2M_N, A2M_N2, A2M and A2M_comp), one A2M receptor binding domain (A2M_receptor) and a Thioester domain (Thioester) are marked on the top of the alignment with a blue line. The signal peptide sequence at the N-terminal is in italic and the putative thioester motif (GCGEQ) is highlighted in black. The thioester associated histidine (His) is indicated by an arrow at position 1991. Six cysteines located at 1392-1530 in the C-terminal are highlighted in yellow.
