# Supplementary material for: Thioester‐containing proteins in the tsetse fly (Glossina) and their response to trypanosome infection
Source: Insect Mol Biol. 2018 Mar 12;27(3):414–28. doi: 10.1111/imb.12382 (PMC5969219; doi:10.1111/imb.12382)
Supplement: Supplementary file 5 — Figure S2. G. m. morsitans thioester‐containing protein sequences. [file IMB-27-414-s005.pdf]

>GMOY001989:GMOY001989-RA cdna:TEP4 KNOWN_protein_coding

gaatgagcaaagaaacctttaaatcaataaagaatatattaaaaagtgagtgaggaggaagaaatacctatgcttcta
ttgtttttctcactaatgatacttgtgttgctaaatattaaacatgggcgttgaccgcatttaagctttccaagaaag
caaaattaatggaactcaacaacaatataaagaccatccgataatcaattgatccttgtgatctttatacgttttaaa
aattttaccacctatcagaatATGTTTACAAAACATTTGTGCCTTGGGCTGATATGGCTAATAACTATAACAGCTACT
GTTTCTCAGCAACAACTACAGCAACAACAACAGCAACAACAACAACAAGATAATTTCTATCAACAAAATCAATATTCC
ACGCCCAGCAACTATGATGATCCCTACAATGGCGCCAATGCAAATTTGAATAATTTTAATCAAAACGATCGCAATCGC
TATCGAACCGATAACGATTTGGATAAGGAGCCTAGTCTGGCGAATCGTGGAAAAACTTTGTATAATAACAAAAATGCC
TTTTTGGAGATGTATCATGCCAAGGAGCCTACTTACTTCATAGTGGCGTCTCGTATGGTACGTCCAGGACTTATTTAC
CAGGTGGCTGTTAATATACTGCAGGCTCAATATCCTATGACCATTCATGCTGGTATATCCTGTGATGGTGTACAAATT
AGTGGCGATTCTAAAGATGTTAAAGAGGGTATACCAGAAACTTTGCTAATGCGAATACCGCCTACTAGTGTCGGCGGT
GATTATAAATTGCGCGTGGAAGGTTTCTATCAAAACGTATATGGAGGTTTTGCCTTCTTGAACGAGACATATTTGCAA
TTTTCCCAGCGTTCTATGTCTATATTTATACAAACTGATAAACCCTTATACATGCAGGGTGAAACCGTACGTTTTCGC
ACAATACCTATCACAACCGAATTGAAAGGTTTCGATAATGCTGTTGATGTGTATATGTTGGATCCGAATCGACATATA
GTACGACGTTGGTTATCTAGACAATCCAACTTAGGCTCTGTTTCTCTTGAGTATAAAATTTCCGATCAGCCAACATTC
GGCGAATGGACGATACGAATTATAGCTCAGGGGCAACAAGAAGAAGGTCACTTTACTGTCGAAGAATATTATCAGACT
CGTTTTGAAGTTAATGTTACTATGCCAGCACATTTCTTCAACACTGATCCTTATATCTTTGGTCGTGTTATGGCCAAT
TTTACTAGTGGCGCTCCCGTACGCGGCAATCTTACTTTGAAGGCGACTATACGTCCCATTGGTTACTTTGATAATCAA
GCTCTGAACGAAAAAATTTCGTTTGGGTAGATCATCTTTCCGGTTAACAAATGCGTATTACGATCAGAATCGTTACAAT
ATTCAATACAATCCAAATTCACCTGAGCAACGAGAAGAGGATAGAATAAGAGAAGAGCAATATTATCGTAATCAATAC
GTGATAGAGAGACTATATCAATTTGATGAAGAGTGGCCATTTTGGATAGCAAAGCCGGATGTACAAGAAACTTATGAT
CCTTGGACAGGGGATTATCGTAAAACACTGCCTTATTTACGGTATTTTAATGGAACATTCGACTTTAAATGGCCAATG
CGGGAACTGGAATTATTAGCACCCAATTTAGCAGGAATGGAAGTCCTGATAACAGCTACCGTGGGTGAAAAGTTTTAT
GATGAAATAATTTCCGGCTATAGTATAGCACGCGTTTACAATTCTAGTATAAGGGTAGCTTTTCTTGGTGACAGCCCT
CAAGTTTTTAAACCTGCTATGCCCTTCACCACGTACCTTGTGGTTGAATATCACGATGGTTCGCCATTGGATGAATAT
TCCTTAAAGCATTCCGGCATTATGGAAGTCAGCGGATTTGTGGAGAGCAAATCTGGAGGACGCAGAGAGTGGCCTGCT
CAACGACTAAATATGAACCCGCAAACTCCAGGTGTCTGGGAAGTAAAAATTGACTTGCGTAATGATTTGCAGTTAGAT
GATCGAGCCCAATCCAGAGATTTTTTGAATGGTGTTACACAAATGCGTTTGCAGGCGAACTTTGTAGACTCGAGAGGC
GAACGTGCTCAGAAGGATTTGTTGTTAGTAGGCCATTATTCTCCTCGCAATCAACATATCAAAGTAACTACGAGCACG
GAGTCCCCTACTGTGGGGGAATATATCATTTTTCATATACGCACTAATTTTTTCTTGGAAGAATTCAGTTATCTAATT
ATGTCTAAAGGTGTTATATTAATGAATGATCGGGAAACGATACGCGAAGGTATCCGCACCATTGCGGTAGTGCTAAGC
GCGGAAATGGCACCGGTAGCCACCATTGTTGTTTGGAAGATTACCCAACAAGGTCAGATTGTTGCCGACTCGCTAACT
TTTCCAGTAAATGGTATTTCTCGAAATAACTTCACTGTTTACATTAATAATCGCAAGGCAAGAACGGGTGAAAAAGTC
GAGGTGGCTATCTTTGGGGAGCCAGGTTCCTATGTAGGACTTTCGGGTATTGATAGTGCTTTCTACACCATGCAAGCA
GGCAATGAATTGACCTACGCGAAAATTATCACAAAAATGTCTAATTTTGATGAGCAAACAAATGGCACATATAAGCAC
ATTTGGAATTCACATGCTGGAAATCCAGACGAATTAATTTATTTCCCTGCATCATCCTTCGGTATAGATGCAAACCGC
ACTTTTGAGTATAGTGGCTTGATTGTGTTCACGGATGGTTATGTACCGCGCAGAGGCGAAAATTGCAATCGTACTCTA
GGTTTTGGAGAATGTTTATCGGGTCGTTGTTATCGCTTGGAAAAACAATGTGATGGCTTATTTGACTGCGACGACGGT
ACTGATGAAATTGGTTGTCGGGCTCGTAACGACACTGATCTCCTAAATTATCGTAAATACCGCTTCAATCGCGTACTG
AGACATTATGAGAATGTTTGGCTTTGGAGAGATGTAAATATCGGACCACACGGACGTTATATTTTTAATGTTGAAGTG
CCTGACATACCTGCTTATTGGATGGTTAGCGCCTTCAGCGTAAGTCCTTCCAAAGGCTTCGGTATGTTGAGCAGGGCG
ATCGAATACGTGGGTGTACAACCGTTCTTCATTAACGTTGAAATGCCCGATGAATGCCGGCAAGGAGAGCAAGTAGGC
ATAAGAGTGACTGTTTTCAACTACATGATTACACCTATTGAGGCCACTGTCGTATTACACGATAGCTTGGATTATAAA
TTCGTTCACGTCGAGGAAGAAGGTATTGTTCGGTCTTATAACCCTCGTACATCATTTGGAGAACATCAATTCTTTGTA
TATTTGGAAGCCCAGGACTCCACAGTAGTTTATGTGCCTATTGTGCCGCAACGTCTTGGCGATATACATGTTACGTTA
CACGTTGCTACACTTTTAGGCACTGATGAAATTACGCGGAAGCTTCATGTAGAGTCGGACGGTTTGCCTCAGTATCGC
CATCAATCAATTTTACTGGATCTTTCAAACCGTGCTTACGTCTTCGAATATATGCATGTAAATATTACCCAGACCCCG
GAGATTCCCTACCAAGTAGACCGATACTTCGTTTACGGGTCAAATAAAGCACGTATTTCGGTAGTGGGTGACGTCGTG
GGTCCAATTTTCCCAACGATGCCTGTAAATGCTTCTTCATTGCTTCATTTGCCCATGGAATCAGCCGAACAAAATGCG
TTCTCTTTCGCCGCAAATGTTTACACTCTTCTATATATGCGCCTGATTAATCAGCGAAATAAGACTCTGGAGAAACAA

GCTTTTTACCACATGAACATCGCTTACCAACGTCAGTTGAGTTTCATGAAACCGGACGGATCATATGCTTCATTTCGT
TCGGATTGGAACAATTCAGATTCCTCCGTGTGGTTGACCGCATATTGTGTGCGCATTTTCCAAGAGGCGTCATTCTAC
GAATGGGAAAACTTTATCTATATAGATCCTACTATCATTGAGAAAAACATACGATGGCTGCTGCAACATCAGACGTTT
GAAGGCGGCTTTTATGAAATAACCTGGTCGCCGGATCGCAAGATGAATCGTACCAATTTCGCAAACCATACCTTTTTG
CAAAACCGTAATATTACTTTAACGTCGCATGTTCTAATTACATTGGCTACAGTTAAAGACTTATCAGGCTCCCTAGGA
GCACGAGTAGCACTGGCTCAACAGCGCGCTATTTCTTGGATTGAGCGCAATATGCAGTTTTTACAAGACACCAAGGAG
CCGTATGATGTAGCAATTACCGCTTATGCTCTACTATTGTGCGGATCGCCCATGGCTGAGCATGTTTTCAGTATATTA
AGAACACATGCTCGCATTGTAGGGGACTATATGTACTGGGGTTACAAAGAATTACCTGATCCGCCAAAAAAATTAGAA
AATCAAAAATTGTTTTCACTGCCCAGACTGCCGTACGAGTATGATTCAGTGAATATCGAGACCACGTCCTATGCTTTG
ATGGTCTATGTATCACGGCGTGAATACGTGGTAGATCCAATAGCTCGCTGGCTAAACGCTCAGCGTTTGTATGACGGT
GGTTGGGCTTCAACTCAAGACACCAGCGTAGCGTTACGCGCTTTAGTGGAATATACAGTGCATTCCCGTTTACGTGAA
GTATCTTCACTTACAGTGGAAATAGAAGCCTCGTCGGCGGGAGGTAAACCAAAAACTTTGCATATCGATGACACAAAT
TTGGCACAATTGCAATCGATTGAGATACCTGACGCTTGGGGCACTGTTAAAGTGCAGGCCAAAGGTACCGGTTACGCC
ATTTTGCAAATGCATGCACAATACAATGTTGATATTGAGAAATTTCAAACCAAGCCTCCTGTGCCTGCATTCGGTTTG
TATACTAAAGCTATTTTCCATGGCCGAAATCAGTCACATATTTCATATCTAACGTGTCAAAACTGGATCAATATACAA
GAATCCGAAAGATCGGGCATGGCGGTGTTAGATGTCACCATACCTACGGGCTATTGGATACAGCAGCAAAAATTGGAC
TCTTACGTATTAAGCAATCGCGTACGGAATTTAAGACGAGCCAAATATATGAACCAGAAAGTGATTTTCTACTTTGAT
TACTTGGACCAGGAGGATATTTGCATTAATTTCACTATAGAACGTTGGTATCCTGTGGCGAATATGTCTCGTTACCTG
CCCATACGAGTTTATGAATATTATGCGCCTGAACGTTTTAATGAAACCATATTTGATGCATTGCCTACATATTTATTA
AACATATGCGAGGTTTGTGGCAGCTCGCAGTGTCCATATTGCTCGATATATAATGTAGGCTGGCGTGCAGGCATGTCC
CTATTTTTGCTCTTCTTTAGTGCATTTATTTCTCTAATGCGCCATCATAATCATATTTCCATCTTAAATATGTTGAAA
TTAATGACATTCTAGtgaaacttgaactctacactttctttttttatatatagtatcttccttactctatcattgtcac
ccaagtgttataagtcaaatgttaaccaaacataattttacattgcttgctaaatgaacaagaatccgtccattatta
cattaatactaagtacaaaatgtagcaaagatttttataaccataaatttttagagtgaaccccataaagaatgaatt
tgcgaaattattcagaaaaacacatagtgtatttctgtaaagaaagtaactgaagcaggatgtatgtaaatgtctcaa
aagaaaacacgaataaaaaatgtaatgttttagaagtgaatttcagatcaaaacttaattttgtgttacattttaaaa
aacgctttgttgaaaattggttttataatgaaaactttttatttgaataaatttttcttttttagtttatatgcttatt
ttaagtctatgtattttatatcattaatttaaaatgaaatgaatgaataatgcgaatttgaaatacaataaattatta
gaatccgtccttcccgacttatgtggcctt

>GMOY010998:GMOY010998-RA cdna:TEP2 NOVEL_protein_coding
acaagtgtaaaataaatttgagtgtgaagaagcacgaatgtttataataaaaaaatttaagaaatttaaaattcactt
ttaaatgaatcgttttctgataaataaaaaaataacaaaataaaaacaaacaATGTTTTATACAAAATATAGTTCACT
GACATTAATAATAATATTGGTGCATTTTTGTGCTGGTCAAGGATTGTATTCCATAATAGCACCGAATACTTTGAGGCC
CAATTCACAATATCACGTGGCAGTTAGTATACACAAAGCTTCAGAGCCGGTAAAAGTCAAAATAGGTATTTTAGGCAG
CACTTACAGCGAATCGAAAACAATCGAAGTGCGGCCCTTTTCAACGGAATTAATAGAATTCGAGATACCTGCTCTTAA
AAATGATCGTTATAGACTTGTAGCCGAAGGTCTAACGGGTATAAATTTCGCAAATGAAACCCAATTGAATTTTGATCA
TAAGCAGCATACAGTTTTGGTGCAAACGGATAAAGCCATATATAAACCGACTGATTTGGTGCAGTTTCGAATCTTAAT
AATGGATGCAAATCTTAAGCCAGCACGCAATTATCCTAACACTCATATAACCATAAGAGATGGTGGCGATAATATCAT
ACGTTCCGATAGAGATGTGCACATAATAAGCGGTGTTTATGCGAACGATTTGTTACTAGCAGATTATCCGAAATTTGG
TGAATGGTCCATAGAAGTGCAAGTGGGCGATGAGATCTATAAAAGATCATTCGAAGTTGTCGAATATATTTTACCAAA
ATTTGTGGTCGATATCACTACCGAGAAGCATGTTATCTATAAAGATAATAAAATCAGTGCCAGCATAAAAGCTTACTA
TATGTTCGGTAAACCTATTGTGGGTGAGGCTACACTTTCTATATATCCCACTTTCTTCGGATCTTTGCAGCCATTCGT
AAACGATTTAATAACACGTAAAGTGATGCCTATCGATGGAAGCGCTTATTTCGAATTCGATATCAGAAACGAATTAAA
ACTTAAAGAAGACTACGAACGTGAATATTTATTAGATGCTTTAGTTGAGGAAAGATCCACGGGATCCGTGCAAAATTT
TTCCACTATAATAACCGTGCATTTAGATCCGTACAAAGTCGAAGCCATTAAAATGCCACGTTACTATATACCGGGAAT
ACCATTTGATGTGACGGTGCGAGTTACGTCTAACGTTGGTGATAGAACAAAAGATTTAAAATCTCAACTTACGGCATA
CTTGACTAACGTTTATGGCAGCAGCGAGATATATAATAAAACTGTTTACAATTTGGATGAGCAGTCGGAAGTTAAAAT
GAAATTCACCGTACCAGCGGGTGATCGAGATGAATATCATTCAGTTATTGTCGACTATATGGGTATTATTACGGATAT
CGGCAAAGTGCCAAGTAAATATATTGGAGGAAAAAATTTTATCATCTCAAAAATCATTACAGAGAAGCCCGTAATAAA

TCAAGAGATCGGTGTTATGGTTCGTTGTAATGAACCTATAAAGTATTTTATATATCAATTGGTTGGTCGGGGCGATAT
CTTATTATCACGCTCCGTAGAGGTTTCCGATAGCACACAGTACACTTTCAAATTCTTAGCTACGTTTGCTATGATGCC
GCGAGCCAAGCTATTAATCTATACAGTGATTAATGGTGAACTGGTTTATGATGAACTGGATGTGGAGCTTGAAGAGAG
TCTTTTAAATAACGTACGAATTGAAGTTCCTTCAACAGCTTCGCCTGGCCAGGATATTGATATTTCCATCGCAGCGAA
ACCTTATAATTATATAGGATTAATGATCGTTGATCAAAATGCTATCAACTTGAGAGAAGGCAATGATCTCTCCGTAAA
GAGTTTAATGAGAGCTTTAAATGATTACGAGCTGAGCGATGTAAATACACCGATTAGCACACCGGGCAAATTGTCGGG
TGTAATTACTCTGACTAATGCGGATTTCTGGCCAGTACAAGAAAGTTTTACAACTATGGAAAATCCTGCATACCATGA
GGACCACAAGCTAACGACTATAAGAAAGACTGATATTGGCCCCGCTCACACGATAGAAGTAAATACTTTAGCCCCCGG
GAAAGGAAGATTTGCTTTTTCGTATACACCTAAACCTTTCTGGCATAATCCGCGTGTACATGTTATGCACCCACCACA
AAATACTTGGCTATTTTTAAATGTAACCGCGGGCAACGAGGAACGCACTATTATACGGGATCGTTTACCCAACGGTAT
GACCAATTGGATTTTAACAGCTTTCGCCATAGACCCAGTGCAGGGCATAGGTCTTGTGCAGCCATCAAAAGTATTAAC
AACTTCCAAGGAATTCTACATAACGTACGAACTGCCTTACTCTGTTAAACTGGGAGAAACGGTAGCTTTGCCATTTGT
GGTAATCAATAATATGGACGATGACTTGGAAGCAGACATTACTTTTTACAATACAGCTCAGGAATTTGAGTTTCCTCA
AGTTACTGAAAAGCCGACACCAAAATCAAAAGTAGAGCTTTACAGTCGTCGTACTTTAGTGGTCGGAGCTAAATCGTC
AAAATCTGTAGCATTTATTGTTACACCGAAGCGGGTTGGTTCTCTTACAGTGAAAGCGGTAGCTGCCAGTAAACTGCT
TACTGATAGCGTAGAGGGTACTCTCTTAGTGGAATACCCAGGATCCACAGAGATAGTTAATCGGGATATTCTTTTCGG
TCTGGAACCTAGCGACAAACGTAATATTAGTGTCCCTGTGCGCATACCACGCAACTCAATAAAGGAGTCAGCTAAAAT
TGAAATATCTGTCTACACGGACTTAATGGGCGGTATTCTTGATAATATGGACAACCTAATATATCAACCTGTTGGTAG
TGGTGAACAAGCCATGATCAAATTCATGCCACATCTTATGGTGCTTAAATATTTACAGCGTTCCAGAAAACTTACGCC
ATCATTGGAAATGAGTGCTAAAAACAATCTAAAGTTAGGTTATCAACGTTTACTTTACTATCGTCATAGAAATGGTGC
CTTTAGTGCCTTTGGGCTAACGGAAGAGAAAAGCTCAACTTGGCTAACTGCCTATGTAGCGAGAGCCTTTAGCATGGC
TTCGGAATTTATACAAATTGATGAAAGCATTTTACAAACGGCTTTAAATTATCTTACCACCGTACAAAATTCGAAAGG
CGGCTTCGACGAACGAGGCGACGAATTCGAACAATTCAGAGATAGTGGCATAAGCTTAAGTGCTTTTGTTACTTTAGC
GTTTATGGAAAACTTGGGTCGCTACTCCTCTTACAAAAATCATATCAACAAAGCGCTCGATTTTGTAACACTTAGTTT
AGATGCAACGGATAATTTGCATTCGTTGGCGATAGGTACCTACGTTCTAACAAAGGCAAATCACAATGCTAAAGGGGC
TTTCTTGCAGCGTCTAGATGCTATGGCGGTAAATGAAGATGGCAAAAAATGGTGGAATAAAACTGCTCCGACTTCTGA
CGCGAATTCGCCCTGGTACAATCGGACACGCAACGTTAACGTTGAAATTTCGTCGTATGCCATTCTGGCTTTGCTGGA
AAACAATCTACTTAATGAGGCATTGCCGGCCATTAAGTGGCTAGTGTCTCAACGTAACGATTTAGGTGGCTTTATAGG
ATCTCAAGACACCGTTGTAGCCTTACAGGCCTTAATCAATTTTGCTGAAAGATTTTCTAGGCCAACCGCTTATTTACA
ATTATTCTTCGACTATGGAGAAAAACCTTCGGAAAAAACAGTTATCGCTGATACTGATAGTACGCTACAAAATCATGA
AGTTCCCAATAACATAAGGAATATGACAATATCTACAGTCGGTACGGGTATGGCCTTGGCTCAGGTAACCTATAGATA
TAATACGTATGTTACTAGGGCTTGGCCACGTTTTGTTTTGGATCCGACCGTTAATAGAAATTCTCATTCCGATTATCT
ACATTTGTCAGCGTGTACGAGTTTTATACCGGCAGAAGGAGGTGCTAATCGTTCGAATATGGCTATTATGGAAATCAG
TCTGCCCAGTGGTTTTGTAGTAGACACTGACACCTTACCCACCCTGGAATCAAGTGATAGAATAAAGAAAGTTGAAAC
TCAAAAACGTAACACCGTAGTGATTATACACTTTGACTATTTGGATCGTAAGGAAGTTTGTCCTACGCTCCACGCCTA
TAAAACCATAAAAGTAACCAATCATAAACCGGTAGCAGTTATAATGTATGATTACTATGACAATGTGAGGCGAGCTCG
TCAATTTTACAGAGCGCCAAAATCACAAATCTGCGATATCTGCGAATACGCTAACTGCGGAACAATGTGTCAAAAGGC
TGAAAAACGTGAATCCAATGGAATAGACGGTGATATTAATGATCTCTTAAATGAAACTAGTAACGCTTTAGGACTTGT
AAAAATTCGAAATTCATATTTAACAGTAATTAATGCTTTGTTAGCATTACTAATAATTAAAGTTTACACTTAAttaaa
aaagaaacaaaaaaaaacaaagatttaaaggaagaatgtattcgaaagaaaaagagaagaaaaagtgtttatagtatt
aatgtaaatggaaaaaaaacaaaaataaagtgacatataaaatactaaactataatgataaactttaaaaatagtaaa
actctgaaaagatattgttattggagaattctcaaaatatgaaatattaatttatgtataaaattttaaataaattt

>GMOY008955:GMOY008955-RA cdna:TEP3 KNOWN_protein_coding
ATGAAATTATCATTTATAGAAGACCTTGAGATATTGATAGACACATCCACCGCTGGTTTGTACATCACAGGTTCCTTT
TTTTTGAAGTACAGCGTTATCGGTCCTGGCACTATACACTCGGATGGCAAGTATACTGTCGCTGTGGCTGTTCATCAT
GTTGCCGAACCATGTCAAATTCAAGTCGGCCTAACTGGGCCCTCTTACAACGACAGTAAAATAGTAGAATTATCGGGT
TTTGAAGTGAAAAATGTTGATTTCGATTTACCCGTTCTGGAGAGGGGTGATTACAATCTGACAGCAGAAGGTCTCAAC
TGTATGAAAATGTTTAAGAATTCAACGAAATTGAATTACAGTAAATTTCACACTAACGTGCGAGTGCAAACAGATAAG
GGTTTATATAAACCAGGCGATGTAATCAACTATCGCGTTATATTTTTGGATAAAAATTTAAGGCCGGATAAACCGATA

AAAGAAGCTAAGATATATGTTGAGGATGGTAAACGTAATCGAATTAAAGAAATCAAAGATTTTAATGTCGTGCAAGGG
GTTTATACCGGCAAATTTCAAATTTCCGAATATCCTGTCACTGGCGGCTGGCGTCTGGGTGTAAGCAATGGTGGCCGT
TATGATCATATGGTCTATTTTGATGTAGACAAATACGTGTTACCCAAATATGTTGTAAAGGTCGAGTCCACCGAAAGA
GTTTCCGTCAAGGATGGCGACATGCAAGTAATCGTTAGAGCTAACTATACCTATGGCAAACCTTTAAATGGCAAAGTC
ACTCTGCTAGTTAACCTTAATGTAAATCGCTACTACTATCGTGGCGATAGTGAGACTGAGGAAACACCAAAAAATCCA
CCGACCATAATCAAAACTGCGCCTATGATTCAAGGCAAATCTAAAATAGATCTCGATGTTAAGGAGTATGAAGCATTT
ATGGATTCAAAGACGTCGCCCAGTTACTTATCGATTGTAGCTACAGTTGAAGAAGAATTTACCGGCGTGAAAATCAAC
GCAACCAGCGGTTCCACTGTGTATCCTTACCGTTATTCTATGAATTGTATTAGTTATGACACATGCTCGGTCTTTCAA
GCCGATAAGGAAGCAGAAGTCGAATTTCAAATAGTTTACGTGGATGGAACTCATCTTAACGATACCAAATCACCGGTT
GAGCTGATTTATACAGAAGTTTTGAATAAATATCGTGTTTGGTATCCGGATTCTGATGAAGAAAATTCTAAAGAGGCT
GATACTGAACCTGTTTCTGAGAATCGCACATTCCATTTTAGAAGTCACATGAATGAAAGCAGTATAGCAGTATTTAAA
GTCTCACTACCGGATCTAAGAGATTATCGAAAGCATGCTCATTTTTATAAAATGGAATTAAAATACCGGGATGAACAA
CGTGAACTGTACAGTACATACCAATACAGAGAACCGAAAAATCTGGATCCCTTATCAGCAGAAGAAAATGATAAACTT
AAAGAATTTTTCCAACTGGAGTACAAGCGTTATGATGACAAGATAGAAATAAATAAAGAAAGTCAATTCACTGTGAAC
TCCAGCCAGCCTTTGTCGTACGTCGTGTATAATGTGGTAGGACGTGGTAATATTTTAAAAAGCGATCGCATCGACTTA
CCGGACAAACCAAAATTCCATAACATTTCATTGACCCCAACCGAAATGTGGGCTCCAAACTTTGCTTTATACGTGTAT
TACGTAGATGAGAAGGGTGAATATCACTATGCTGAACAACGGTACTATGTTCAATATAGACTGCAGAATCAGATTAAT
ATAACTGCACCCGAACAAGTAAAACCCGGTGAAAATGTATCATTGAAAATAAAAACAGCTCCCAACTCATTTGTAGGT
TTGACAGCAGTTGATCAAAGTGTTTTATTGCTAAGGTCTAACAATGACTTACGTCCTCACGAATTCGACTGGGTTTTG
AGTAGCTACACCACAACAACTCCACACCAAGGTGGGTATTCAGACTATCCAGGTTGGAGTAGCGGAGTGGTGACCTTA
ACCAACGCTGACTATTTCTATAATTGGACCAAGCCGGAGTATCTTTCGACTCCCCTTTCGGCTCAACTAGACTCAGAA
CTTAATAATCGAATTTTTACTAAATCTGGTGTACAGGAATCTGGCATTTTGGGAGCTGCGGGAAGACCAGCAATGGCA
GAAGCAGATTCAGGTTCTGGATTTTCCGCTAGTAGCGCTGAAGTACAAGTGCGTAAAGATTTCTCAGAAACGTGGTTA
TTTGATAACATCGAAAGTACCAACGAGGAAGAGTTTACTTATGTGACAAAAATACCCGATACTATAACTTCGTGGCTT
ATTAGCGGTTTTTCAATGAATCCTAATAAAGGTCTGGGCATTACAGCAGACAAAACAAAAGTGGTTACATTTCAACCT
TTCTTTATATCTATCCGTTTGCCTTATTCCGTAAAACGAGGCGAAGTTATCAATGTACCTGCCTTAGTATTCAATTAT
CTAAACAAAGATTTAGATGTCGAGGTCATCTTAGATAATAACGATGGTGAATATGAATTTATGGACATCACTAATGAA
GTACATAATGATGAAAAGCAGGTTAAAAAAGTAGTGCGAGTGCCGGCTCATGGAGCTGCTGGGGTCTCCTTCATGTTA
AGGCCCAAAATTATCGGTAATGTGATGTTAAAATATCTCGCTAAATCTCCGTTGGCCGGTGACGCCATACATAAAACT
ATGAAAGTAGTTCCTGAAGGTGTAACACAATACGCTAACCGAGCTTATTTTGTGAATTTAAAAAAAGAATCAGAGGAG
AAAACCAACTTTAAGTTGGAATTGCCGGATGATGTGGTGCCGGATTCGCAATACGTTGAAGTTGGTGTCATGGGTGAT
CTATTAGGTCCAGTACTTAATAATCTGGATAATTTAGTACGAAAACCAGCAGGTTGTGCTGAACAGACCATGTCCAAG
CTACTTCCTAACTATCTAGTAATGAAATATATGCAGCATATCAACCAATTAACACCCGGCTTGGAAAAAAGATTACTT
TATAACATAGAAAGCGGTTATCAGAATATGTTAAACTTCCGCCTTAAAGATGGTTCTTTCAGTGCGTTTGGTTTACCG
CAATATTATCGCGATGAAAAAAAACCCACTAATGGCTCAACTTGGCTAACCGCCTATATAATACGTTCTTTTAATCAA
CTTAAAGAATTTGTGAATATTGATGAGCGAGTGATCAATGAGGGCTTGGAATACATGGTTAAAAATCAAGCAAAAAAT
GGCAGTTTTATCGACAAGGGAAATTTTTATTATGGTGGTAGTCGAGACGTCATTTCATTAACATCTACCGTTCTATTA
GCTTTCTTGGAAAATAAGACCATCGCCGACCAACATAAAGATGTGATACAAAAAGGTTTAGATTTTATATCTAAAAAT
ATCGATAAACCCAAAACATTAAAAGATCATATTCTTGGCACATATACTCTTCATAAGGCTCAACATCCGCAAGCAGAG
GAAGAATTGATCAAAATTAAAAACTTAGCCAAAACCGAAGGAGATCGAATGTGGTGGTCAGAGTCGGACGATAGACCT
AAAACGTATTACTTCTTCTCGAATGATGTGGAGATAACTGCGTACAACTTGTTAACTCTGCTGGATGAATCGTCCACT
ACAGTTGATGATGTACTGCCCATCATAAAATGGTTGATAGCCCAACGTAATAGCTATGGTGGCTTTTCTTCTACTCAA
GATACTATAGTAGGCTTGCAGGCTATTATTAAATTCGCAGAGAAGGCTGACTATAAAGCCGCAAAAATGGATATAGAA
ATTGAAGCGAAAGGGGATATGCCCAAAAAAGAAACCTTACATCTAAATGAGGAAAATGGTATACTTTATCAAACGCTA
GAGTTGCCGGCCAAAACTTCAAACATCGAATTCACAGTAAAAGGGGCCGGCTCTGCTTTGGTGCAGATATCTTATCAA
TACAATATCTTCGAAAAAGCTCCACAGCCCAGTTTTAGTATCGATACTCAGAAACATGACAGCAGTTTGTCAGGTAAA
CTGCTAATGGATGTCTGCGTAGATTACATAGGAGAAGGCGATTCCTCCAATATGGCTTTGCTGGAAATTTCTTTACCT
TCCGGTTTTGTTATCGATGAAGATTCGCTTGAAAATTTAAAGCAAATTGAAGGAGCTAGCATGAAGCCCTACTTTACC
GTTTTCTTTAAACAGAATATCGAAGTGAAGAATTCTGCCTCTCTTCTGGTTATTTACTTCGATCACCTGCACAAAAAT
AGGCAAAAATGTGTACCCATTGAAGCCTTTAAAAGTCATGCTGTGGCCATGCAAAAACCTGCTTCTATTCTTTTGTAT

GACTACTATGATACGAACAAAAAAGTGACCAGCTTCTATGAGGTCGCGTCCAAATTATGTGATATCTGTGACGGTGAA
GAAGAGTGCACAAATTGTGAGTGGCAACTAAATGCCGCCAAACGCCATCACATGGAAACTGACTATCTATGGATGATG
TCGATTCCGAATTTTCCAGTATTCTTCACCAATGCTATCGAACAGAGCAATCAACTGAAGCGCACTTACGTTAAAAAA
TTGTTGCGCGTCATTGATGACGATGATATCATTGCTGATGATGCTGATGATGCTTCATGA

>Gmm_TEP1

ATGAGCGAGCCAATTTACACTATTGTGGCGCCTGACACCATACGCTCTCATCAGAAATTTCCCGTTAGTGTCACTTTA
CATGACGCCCAGACAACGGTTACTTTGGACATAAGTGTTACTGGTCCATCATACAATCAGTCTAAAATTGTCAATTTA
TCTTCAATGGAAAACAAGCAAATAGATTTCGATGTACCTGCCTTATGCGATGGTTCGTATCAACTGACTTCTAAAGGC
ATCGAAGGTTTGCAAATTGAAAAGTCAACCGCTTTGTATATGGATACCAATCAACCAAATATATATATACAAACGGAT
AAAGCCGTATATAAACCAGGAGATTTAGTACAATATCGCATTTTAATTTTGGACGAGAATATCCGGCCAGTGAAATTG
GAAAGACCTCTAAGAGTGGCAATTAAAGATGCAGCGAATAATTACATTAAAGAATTTAAAGTTTCCCACTTGACCAAA
GGCGTTTACAGTGGTAGATTTCAATTAACGGAACAACCCATATTAGGTCAATGGACAATTGAAGTTGATTTAGCAAAG
GACGCACAACAAGAAAAGAACTTTAAAGTCATTAAATATGTGCTACCTAGGTTCAGTGTGGATATCGAAACAGTTAAA
GATTTGGCTATAAATGACAATTCCTTAAAAGTGGCAGTTTGTGCAAAATACACGTACGGCAAACCGGTCAAAGGCAAA
GCTATCATAACCATATTATATTTAAATCTTGAGAAAATCATTGATATAAATGGTAAAGAAGAAGTGGAGTTTACTTTG
CACAAAGGCTTGAATTGGAACTCTCTAGGCGAAAATATGACAATTTCCGCTATAGTAGATGAGGAACTAACGGGAAAT
CGGCAAAGTAATACTATCGATATAAAACTTCATAGTTCTCAATACGTTGTCAAAATGCTCGACTCGGTGATTGAATTC
GAAATCAATGAACCGTTTGTGGTGAAGGCAGCTGTGGAATATTTGAATGGCGATCCCGTCCGGAATGCAAAGGATCCA
ATCTTTCTTAAGTATTATAGAGGATGGGGGGAACCTGAAGAATCGCAATTATTTGAGAGTACTTTAGATGATAATGGT
GTGGGAATGTTTAAAGTCAATTTACCCAATGGTGGCATTTATCTGGGCGAACTTAGATTCATGGATAAAGTCGAAATT
TTGCCGTGCATTATGGCCAAATCACATAATACAACTTTAGCTCAAGCTGAAATAAGAGAGGAATTGACTCTTGTGTTA
AACACAGAAAGGCCGCGAATAGGTGATGACGCGTCTGTAACTGTAAAAGCACCTAATATGATGACTCATTTGACATAT
GTAATTGTTGGTCGTGGTTGTATTCTACAAACGGCTTATATTACTTTGCCCGAGCCGGCAAATTTCTATAAAATCAAC
TTTAAAGTTACATTCGAAATGATTCCACGCGCTGATGTGTTCGTTTTTTACGTGGATAAGAGTGACCTCAAATATCAA
GAAATCAGTATTGACTTTGAATTGGAATTTCAAAACAGTATAAAACTAACAGGTCCTCTGCAAGCGAAGCCTGGACAA
GAAGTTAGTTTGGATATCGAAACCGTTTCGAATTCGTTTGTTGGCTTGTTGGGCGTTGATCAAAGGACTTTGCTTTTA
GAGAGGGGCAACGACTTCGAACGGAATACAATATTAAATAATTTAAGACATCATAATACTAATATTGGTTTTTTCCCC
TATCCGGGTAAAATGTCTGGCTTGGCAGTACAGACAAATGCTCGTTTTCCTTACGAAGAATTAGATCGACATTGTATT
TTATATTCACAATGTATGCAGTGTCAAGCAGCTCTTCCCGCACAGGAGGTTTTCGTCCAAAGAATTCGAAAAAATTTT
GACGAAGTCTGGTTGTTTGAGGATATTGATAATAATGATAATACAACAAACGTGACAGTAACTAAGTGCATACCTGAT
AGCATGACTTCTTGGATAATAAGCGCATTTGCGATAAATGCGAGAACTGGTTTTACAATGACCGAGAATCCTTTGAAA
ATTAATGTATTTCAGCCCTTCTTCATCGATGTAAATTTACCGTATTCTGTAAAAAGGGGCGAAGTTATTGAAATACCA
GTCGTTATTTTCAATTACCTGAATAACACGTTAGAAGCCCAAATCGTTATGGAAAATACGGACGGAGAGTATGAATTT
ACCGATGTTTCAAAGGAAATTGAAAAGTCTTCATTTATTGCTCAACGACGAGCTAAGAAAGTAATGGTGTCCCCGAAC
AGCAGTCAAAATATATCTTTTGTAATCCGTCCACAAGTAATTGGAGATGTAATGTTAAAAATTGAAGCCATTACTTCG
CTGGCCAGCGATGCTATACATAAAAAACTAAAAGTAGAAGCTGAAGGTGTCACCCAATACAAAAATCAAGCTCTTTTC
TTAAATTTGGAGAAGCCACAGGAAGCTTCGCTAGAAGTCGCTATACCCACGGAAGCTGTTAAAGACTCGGAATATGTG
GAATTCTCTGTTGTTGGTGATCTCTTGGGTCCCACTGTCAAAAATCTTAATGAGCTTGTACGCAAGCCCTACGGTTGT
GGTGAGCAGAATATGGTTAATTTCGTGCCCAACATATTGGTCTTACATTACTTGGAAGCCATGCAAATTGATATGCCA
AATGTAGCGAGCAAAGCTAAAAACTTCTTAGAAATTGGTTATCAACGTGAGTTGACCTATAAGCACAAAAACGGCGCA
TATAGCGCTTTTGGCGAAGATAGAAGCACACCGAATACTTGGTTAACCGCTTATGTGGCTCGTTCCTTTATCCAGGCA
TCTAAATACACGACCATTGATGAAAATGTTATCCAACAAGCTTTCGAGTTCCTAATTGCTAACCAGGAGAAAAGCGGG
CAATTTAAGCAAACTGGTCATCTCTTCAGTCCAACTCATCAGAATGATGTTGGATTCAATGCTTATGTGCTGTTGGCC
TTCCTAGAGAGTGAAAAATACGCGGAGGTTTATCAAAAGCAGATAGAAAAATGCCTCGAATACGTAGTAAGCCAGCTG
GAGAATGAAAGGGATTCTTACGCTCTATCTATAGCTGCAAGTGCTTTGCATAAAGCTAATCATAAGGCTGTGGGTGGA
GTGCTGGAAAAGCTACAGGCACAAGCCAAGGAAGAAAACGGTTTCAAATGGTGGACTAGTGCGAACCATAATGATATT
GAAATCACTGCCTATGCGTTGCAAACTTTGGTTGACACGGAATCCACTAATCAAATTTTACCAATTATCAAGTGGTTA
ATCGGTCAACGTAATAGCAATGGTGGTTTCGACTCTACCCAAGACACTGTAGTAGGTCTTGAGGCACTTATTAAATTC
TCTAAGAAATTTTCAATTACCGGGAACAGCATAATGTCGATAACATTCAAAGCTTTTGATGATGGAAAGAAGGAACTT
AGCCAGCATAGATTTGAGGTGGACAAAGACAACTCATTGGTCTTACAAACGCACGTGTTACCGAAATCCACTCGCTCA

TTAAGTTTGGAAGCTGATGGTGTTGGTTCTTCGTTAATTCAACTTTCCTATCGATACAACTTGGCAACTAAAGATGAT
ACGCCCAGTTTCAAACTCGACATTAAGCCAAAAATTTTACCATCGCAACAATTACAGATTGAAGTTTGCGCCAGCTAT
GAACCTCATGCTAGTGATAAGATTTCACAATCCAATATGGCCGTCATGGAAGTTGCTTTGCCTTCCGGTTATATTGCT
GACAACGAGAAATTTGATGATATACTTGCTGTCGAGCGTGTTGAACGGGTGGACACCGAGAACTGTGATACAAAGGTT
ATCGTATATTTCAATGGCTTAGTGGAAGGCGAAAAAAAGCGTGTTAATATCATAGCAGATAGAGCCTTTGCTGTAGAC
AAGCAGAAGCCGGTTCCCATCATTTTATACGACTATTATAACAGTGAATATCGTGGAACAGAATATTATCAAATTACA
TCTCCGTCACCTGATATTTGCGAAGAAAGTTAA

>Gmm_TEP5

ATGTGGCAAAATCGAATGTATATTCTTTTGCATATGCTATGCGTTGTGAATGCAAACGGAATTTACTCCATTGTGGCC
CCCGGCAGTATATATTCAAATCGCAAATATTCCGTTAGTGTTACATTACACGATGCTGGGCAAGCAGTTACGTTTAAC
ATAGGCATTTCTGGTCCATCATACAATCATTCCAAATCCATTGAATTGTCTCCAAAGGAAAATAAGAGAATCGATTTC
AATGTCCCAGAATTAAAGAAAGGCATATATCAACTTGTCTCGAAAGGAATCGGAGGATTATACTTTGAAAACACTACA
TACTTATCCGTGGAATACACCAGACCTAATCTTTACATACAAACGGATAAAGCAATGTATAAGCCGGGAGATTTAGTA
CAGTATCGCATTCTAATTTTGGATGAAAATTTTCGACCGCTGAAATCGGACCGATCTTTAGGAGTTGCCATTAAAGAT
GCAGCAAATAACCTTGTGAAAGATATTAAAAATGCTGAAAATTATAAAGGCGTTTTTAGTGATAAATTACAACTTACC
GAACAGCCCGTTTTGGGTCTGTGGATAATTGAAGTTAGTTTAAACGATCATATAGAAAAAACGAAGGAGTTTGAAGTA
GCAAAGTATGTGTTACCCAAATTCAGTGTGGATATTGATGCTGTTACAGATTTGGCCATAACTGAAAGTTCTTTAACG
ATAACTGTACGTGCAAAATACACTTATGGTAAGCCAGTCAAGGGTAAGGCTACTGTTCATTTATCGCCCGTAGATTTG
GAGAAAACTATAGACATCAATGGCAAAGGTCATGTGGAATTTGATTTAAAAAAAGATTTAAATTTAATTGTTTCGAAC
CGATTCGTGAGAGAACTGAAAGTGTTTGCAGTGGTGGAAGAAGAGCTAACTGGAAATAGGCAAAATACTACACTCAAA
ATAAATCTTCATCGCTCTCCATATAGAATCGAGGTCTCCGATATGATGAAGGAGTTTGAAATTAATCAAACGATTGAA
GTGAAAGTAGTTATTAAATATTTAAATGGTAACCCAGTCCAGGACACAAAAGCTCCTGTTCTTCTCAAATTTTACAAT
ACAAGGAGAGCAGATGAAGATCCTGAAATTTTTAAGGCAACCTTAGACGAACATGGTGTGGTCATATTTAAAATTAGC
TTTCAAAATGATGGGTTTTATTGGCCTGAACTTAAATTTGCTGAGGAAATTAAGCACATGCCAAGCATTAGCGTAAGG
GCAGCTAGTGAAAAAAATACTAAGTTAGTATCCCAGTTAACCCTTGAGTTGGAGACTATAAAGCCCCGTTTAGGTGAA
CACGTATCGATAGCTGTTAAGGCACCCGATGTTATGAATCATTTGATATATGCGGTTGTAGGCCATGGTACCATACTG
CAAATAGCTAACATTTCTTTGCCGAGACCCCAACAATTTTATAAAGTTACCTTTCGAGCTACATTCGAAATGATGCCA
AGAGCTAACCTATTTGTGTATTACGTCGATGAATCGGATTTGAAATTTCAGGAGATCACTGTTGAATTTCTTCCGGAG
TTTGAAAACAAAATTGAGATAACGGGCCCCATACAAGCAACGCCTGGTCAGGAGGTTTCTTTGGAAATTAAAACCGAT
CCGAACTCTTATATCGGCTTATTAGGAGTAGATCAAAGTATGTTGCTTCTGAAATCTGGCAATGATTTAGAATTCGCT
GCAATTCTAAACGATTTACGTAACCGCAAACCTATAGATAAACATTATCAAAACACCCGTCTTAAAAAGGGAGCTTCA
CATTATGAAATAGAAGAAGAAATGAGTGAAAGTGTAGACGATGATGCCGAAGGCGTAGATGAAGTCGATACCAGCTGC
GTTAAATGCGAGTTTGTCCTTCACGCAGTGTCCAACGTAAACACACGTACAAACTTTCGAGAAATTTGGTTATGGTCT
GCTTTGAATTTTAGTGACGATAATATCACCAGTGCAACAATCACACAATCCATACCAGATACAATAACTTCTTGGGTA
ATCAGCGCTTTTGCGGTGAACGAGAAAACTGGTTTGGGTATGACCGAGAATCCGTTCAAAATTAACGTATTTCAACCA
TTCTTCATCGATGTAAATTTGCCATATTCTGTAAAAAGGGGCGAAGTCATTGCCATACCAGTTATTATTTTCAACTAT
ATGGATAAAATATTGGATGCAGAAATCACCATGGACAATACTGACAAGGAGTACGATTTCACCGAAGTGTCTAATGAA
ATTGAAGAATCTATTCTGAATGCGCAAAAGAGAATGAAACGGGTATCGGTGCCTCCGAATAGCGGAGAAAGCGTATCT
TTCATGATACGTCCAACCGTAGTAGCTGATATAGAATTGAAAATTATCGCTATATCTCAGTTGGCAGGGGACGCTATA
CATAAAAAACTAAAAGTAGAAGCTGAAGGTGTCACCCAATACAAAAATCAAGCTCTTTTCCTAAATTTGGAGAAGCCA
CAGGAAGCTTCGCTAGAAGTCGCTATACCCACGGAAGCTGTTAAAGACTCGGAATATGTGGAATTCTCTGTTGTTGGT
GATCTCTTGGGTCCCACTGTCAAAAATCTTAATGAGCTTGTACGCAAGCCCTACGGTTGTGGTGAGCAGAATATGGTT
AATTTCGTGCCCAACATATTGGTCTTACATTACTTGGAAGCCATGCAAATTGATATGCCAAATGTAGCGAGCAAAGCT
AAAAACTTCTTAGAAATTGGTTATCAACGTGAGTTGACCTATAAGCACAAAAACGGCGCATATAGCGCTTTTGGCGAA
GATAGAAGCACACCGAATACTTGGTTAACCGCTTATGTGGCTCGTTCCTTTATCCAGGCATCTAAATACACGACCATT
GATGAAAATGTTATCCAACAAGCTTTCGAGTTCCTAATTGCTAACCAGGAAAAGAGCGGGCAATTTAAGCAAACTGGT
CATCTCTTCAGTCCAACTCATCAGAATGATGTTGGATTCAATGCTTATGTGCTGTTGGCCTTCCTAGAAAGTGAAAAA
TACGCGGAGCTTTATCAAAAGCAGATTGAAAAATGCCTCGAATACGTAGTAAGCCAGCTGGAGAATGAAAAGGATTCT
TACGCTTTATCTATAGCTGCAAGTGCTTTGCATAAAGCTAATCATAAGGCTGCGGGTGGAGTGCTGGAAAAGCTACAG
GCACAAGCCAAGGAAGAAAACGGTTTCAAAAGGTGGACTAGTGCGAACCATAATGATATTGAAATCACTGCCTATGCG

TTGCAAACTTTGGTTGACACGGAATCCACTAATCAAATTTTACCAATTATCAAGTGGTTAATCGGTCAACGTAATAGC
AATGGTGGTTTCGACTCTACCCAAGACACTGTAGTAGGTCTTGAGGCACTTATTAAATTCTCTAAGAAATTTTCAATT
ACTGGAAATAGCAAAATGTCGATAACATTCAAAGCTTTTGATGATGGGAAGAAGGAACTTAGCCAGCATAGATTTGAG
GTGAACAAAGACAACTCATTGGTCTTGCAAACGCACGTGTTACCGAAATCCACGCGCTCATTAAGTTTGGAAGCCGAG
GGTGCTGGCTCTTCGCTAATTCAACTTTCGTATCAATACAACTTGGCAACCAAAGACGATAGACCTGGATTCAAAGTA
GACATTAAGCCGAAAATTTTGCCATCGCAACAGCTGCAGATTAATATTTGCGCAAATTATCAACCGGCTGTTGACGAT
GAAATCAAGGAATCCAATATGGCTGTCATGGAAGTTGCTTTGCCATCTGGTTATATCGCCGACAATGAGAAATTTAAT
GATATACTTGCTGTCGAGCGTGTTCAACGCGTCGATACCGAAAACTCTGATACAAAAGTGATCGTCTACTTTGATGGC
TTAGTGGAAGGTGAACAAGTATGTGTTACTATCCTGGCAGACAAAGCTTTTGCTGTTGCTAAGCAGAAACCAGTTCCC
GTCACTCTGTACGATTACTACAATAGCATATATCGTGGAACCGAATATTATCAAATTGAATCTTCGTTGTGTGATATT
TGCGAGGGAAGTGATTGTGGAACTGCGTTTTATTGCAatgaaattataataatattataatttgtctccacttaataa
ataaagctctaaattgaagct

>Gmm_TEP6

ATGTTTCAAAACATTTGCTTAGTTTATTGCATTTTACATCTGCTGTGGCTGGTAAGAGGAAGTGGAATTTATTCTATT
GTGGCTCCGAAAACCTTGCAGTCAAACCACAAATATTCTGTCAGCGTTACTTTACACGATGCCAAGCAACCGGTTACT
CTCAACATAGGCATTACCGGACCTTCATATAATCATTCGGAAACTGTGAATTTGACTGCGATTGAAACGACTCAAATA
GACTTTGTGTTACCTGCATTAGACGGAGGTCCGTACAGATTAATTACCAAAGGTATTGAAGGGTTAGACTTTGAAAAT
GCTACAGAGTTACATGTCACACAGAGTACGTCAAATGTTTACATTCAAACGGACAAAGCGATGTATAAGCCGGGAGAT
TTAGTGCAATATCGCATTCTAGTTTTGGATGAAAATCTTCGACCGCTCAAATCGGACCGACCTTTAGGAGTTGCCATT
AAAGATGCAGCAAATAACCTCATAAAGGATATTAAAAATGTTCAACTCATTAAAGGCGTTTTTAGTGATAAATTACAA
CTTACCGAACAGCCCGTTTTGGGTTTGTGGATAATTGAAGTTAGTTTAAGCGATCATTTAGAAAAAACAAAGCAATTC
GAAGTAGCAAAGTATGTGTTACCCAAATTCAGTGTGGATATTGATGCTGTTACAGATTTGGCCATAACTGAAAGTTCT
TTAAAGATAACTGTACGTGCAAAATACACTTATGGTAAGCCAGTCAAGGGTAAGGCTACTGTTCGTTTGTCGCCCATA
AACCTGGAGAAATCTGTAGACATGAATGGCAAAGGTCATGTGGAATTTGATTTAAGAAAGGATTTAAATATAATTCGA
GAAGAATCGGTAAGAGAACTGAAAGCTTTCGCAATGGTGGAAGAGGAGCTAACTGGAAATAAGCAAAATGCGACAGTC
AAAATAAATCTTCATCATTCTCCATATATAATCGAAGTCTTCGATGTGATGACAGAACATGAAGTTAATCAACCGTTT
GAAGTCAAAGTAGTTATTAAACATTTAAATGGCAAACCAGTCCAAGACACAAAAACTCCAGTTCTTCTTAACTATTAT
TATAGATGGGGAGAATATAAGAACGCTGAAATTTTTAGGGCTAATTTAGACGAGCACGGTGTGGCCATAATTAAAGTT
AATTTTCAAAAGGATGGTTTTTATTGGTGCGAACTTAAATTTGCTGATGAAGTTAAACGGTTGCCGAGTGTGGGCGTA
GCAGCAGCTCGTAAAAAAAATACCGAGCTATTAACTCTTGAATTGGAGACAATAAGGCCGCAATTAGGTGAATATGTG
TCCATAGCTGTTAAAGCACCTAAAGTTATGACACATTTGATATATACTGTGGTAGGACGCGGCAACATTTTGCATAAG
AGGAACATACCTTTGCCTAATCCGCAAAACTTCTACACAATAAGTTTTAAGACTACCTTTGAAATGATACCTAAAGTG
CATGTATTTGTTTACTATGTCGATGAGGGCGATCTAAAGTTTGAAGAAATTGGCGTTAAAATCCAGCCGGAATTTGAA
AATAAGATAGAAATAACTGGCCCTAGTCAAGCGAAGCCTGGTCAAGAAGTTACTTTCAATATTAAAACTGATCCGAAT
TCATATGTCGGATTATTGGGAGTGGATCAAAGCGTCTTACTATTAAAGTCTGGCAACGATGTGGATCTCTCTGCTATA
CTCAATGATCTAAAACCTTACAAAAGTGAAGACGAACATCAAGATGAATTTTCTAGATATATTTACAAAGTTCCTGGG
GAAAATTCTGGTTTAATGGTAATGACCAACGCCCATTACCCCTACAAAGATCAACGAGTTGTAATATCGTATGCGATC
CATGGGGCTGGTCCTTACTATTTGCTTTCTTCAAATCCTGAAATTGAAACTTCAAGAACTTTACCTGAATATTTACCA
CCTTCAGCGGGGCCCGAAAGGATACGAAAAGATTTTGCCGAAATTTGGTTATTCGAAAATTTAGATGATAACAATGAA
ACCGGGCACTTGACATTCACCAAACCTCTGCCTGATACAATAACTTCTTGGGTAATCAGCGCTTTTGCGGTGAACGAG
AAAACTGGTTTGGGTATGACCGAGAATCCGTTCAAAATTAACGTATTTCAACCATTCTTCATCGATGTAAATTTGCCA
TATTCTGTAAAAAGGGGCGAAGTCATTGCCATACCAGTTATTATTTTCAACTATATGGATAAAACATTGGATGCAGAA
ATCACCATGGACAATACTGAAAAGGAGTACGATTTCACCGAAGTGTCCAATGAAATTGAAGAATCTATTCTGAATGCG
CAAAAGAGAATGAAACGGGTATCGGTGCCTCCGAACAGCGGAGAAAGCGTGTCCTTCATGATACGTCCAACTGTAGTA
GCTGATATAGAATTGAAAATTAACGCTATATCTCAGTTGGCAGGGGACGCTATACATAAAAAACTAAAAGTAGAAGCT
GAAGGTGTCACCCAATACAAAAATCAAGCTCTTTTCTTAAATTTGGAGAAGCCACAGGAAGCTTCACTAGAAGTCGCT
ATACCCACGGAAGCTGTTAAAGACTCGGAATATGTGGAATTCTCTGTTGTTGGTGATCTCTTGGGTCCCACTGTCAAA
AATCTTAATGAGCTTGTACGTAAGCCCTACGGTTGTGGTGAGCAGAATATGGTTAATTTCGTGCCCAACATATTGGTC
TTACATTACTTGGAAGCCATGCAAATTGATATGCCAAATGTAGCGAGCAAAGCTAAAAACTTCTTGGAAATTGGTTAT
CAACGTGAGTTGACCTATAAGCACAAAAACGGCGCATATAGCGCTTTTGGCGAAGATAGAAGCACACCGAATACTTGG

TTAACCGCTTATGTGGCTCGTTCCTTTATCCAGGCATCTAAATACACGACCATTGATGAAAATGTTATCCAACAAGCT
TTCGAGTTCCTAATTGCTAACCAGCAAAAGAGCGGGCAATTTAAGCAAACTGGTCATCTCTTCAGTCCAACTCATCAG
AATGATGTTGGATTCAATGCTTATGTGCTGTTGGCCTTCCTAGAAATTGAAAAATACGCGGAGCTTTATCAAAAGCAG
ATTGAAAAATGGCTCGAATATGTAGTAAGCCAGCTGGAGAATGAAAAGGATTCTTACGCTTTATCTATAGCTGCAACT
GCTTTGCATAAAGCTAATCATAAGGCTGCGGGTGGAGTGCTGGAAAAGCTACAGGCACAAGCCAAGGAAGAAAACGGT
TTCAAATGGTGGAGTAGTGCGAACCATAATGATATTGAAATCACTGCCTATGCGTTGCAAACTTTGGTTGACACAGAA
TCCATTAACCAAATTTTACCAATTATCAAGTGGTTAATCAGTCAACGTAATAGCAATGGTGGTTTCGACTCTACCCAA
GACACTGTAGTAGGTCTTGAGGCACTTATTAAATTCTCTAAGAAATTTTCAATTACTGGAAATAGCAAAATGTCGATA
ACATTCAAAGCTTTTGATGATGGAAAGAAGGAACTTAGCCAGCATACATTTGAGGTGAACAAAGACAACTCATTGGTC
TTGCAAACGCACGTGTTACCGAAATCCACGCGTTCATTAAGTTTGGAAGCCGAGGGTGCTGGCGCTTCGCTAATTCAA
CTTTCGTATCAATACAACTTGGCAACCAAAGACGATAGACCTGGATTCAAAGTAGACATTAAGCCGAAAATTTTGCCA
TCGCAACAGCTGCAGATTAATATTTGCGCTAATTATCAACCGGCTGTTGACGACGAAATCAAGGAGTCCAATATGGCT
GTCATGGAAGTTGCTTTGCCATCTGGTTATATCGCCGACAATGAGAAATTTAATGATATACTTGCTGTCGAGCGTGTT
CAACGCGTCGATACCGAAAACTCTGATACAAAAGTGATCGTCTACTTTGATGGCTTAGTGGAAGGTGAACAAGTATGT
GTTACTATCCTGGCAGACAAAGCTTTTGCTGTTGCTAGGCAGAAACCAGTTCCCGTCACTCTGTACGATTACTACAAT
AGCATATATCGTGGAACCGAATATTATCAAATTGAATCTTCGTTGTGTGATATTTGCGAGGGAGATGATTGTGGAACC
GCTTGTGAATGAaattataacaatatttaaaaatataaatgtaatcaataaactactacatactatgctagat

>Gmm_TEP7

ATGTTTCAAAACTTTTATTTAGTTTATTGCATTTTACATATGATGTGGCTGGCAAGAGGGAACGGAATTTATTCTATT
GTGGCTCCGAAAAACTTGCAGTCAAACCACAAATATTCTGTCAGCGTTACTTTACACGATGCCAAGCAACCGGTTAGA
TTCAACATAGGTATTACCGGGCCATCCTATAATCATTCGGAAACTGTGAATTTGACTCCGATTGAAACGACTCAAATA
GACTTTGTGTTACCTGAACTAGATGGCGGTCCATATCTCTTAATTTCCAAAGGTGTTGAAGGGTTAGATTTTGAAAAT
GCTACAGAGTTACATGTCACACAGAGTACGTCAAATGTTTACATGCAAACGGACAAAGCGATGTATAAGCCGGGAGAT
TTAGTGCAATATCGCATTCTAGTTTTGGATGAAACTCTTCGACCGACAAAACTGAAGGAAGCCTCTTTAAAAGTGACT
ATTAAAGACGCACAAAATAACCTTGTAAGAGATGCTAAAAATGTCAAACTCATTAAAGGCGTTTTTAGTGATAAACTG
CAGCTTACTGAGCAGCCCGTTCTGGGTGTCTGGAAAATTGAAGTTAGTTTAAATGATCATGAGGATAAGACAAAGACA
TTTGAAGTAGCAAAGTATGTGTTGCCCAGATTCAGTGTTGATATTGACACAGTTACAGACTTGGCAATCACTGAGCGC
CTTTTAAAGGTAACGGTACGTGCAAAATACACTTACGGTAAGCCCGTGAAAGGGAAGGCTACTGTTCGCTTGTCGCCT
GTAAACTTGGAGAAAACTATAGACGTGAACGGCAAAGGTCATGTGGAATTTGATTTACAAAAAGAATTAAAAGCGGCG
GTTAAAGAAGGAGCCGTAGAAATTTTCGCAGCGGTTCAAGAGGACCTAACTGATAGTCGGCAGAATAATACGATTATC
GTCAATTTTCATAGTTCCCAATATATCATCAAAACACCCAATGCAGTGGGAGAGTTTGAACCCAATAAACCATTTGAA
GTAAAAGCAGTTATTCAATATTTAAATGGCAAACCAGTCCGAGATGCCAAAACTCCAGCACTTCTGCATTGTTACCAA
GGGTGGGGGGGAAATATTAAGTCTGAAATATTTAAAGCTAAGTTAGATGATCACGGTGAAGCTATATTTGCGGTCAAG
TTACCACAGGCAAGCGTTTTTCGATGTGAAATAGAATTTGCTGATAAAACTGTTCATCTTCCTCCTATTCATCGAAAA
ATAGACGAAAATAATTTTGTTAAGATGACCCGGGAACTAACCCTTACATTGGTTGACAAAACTCCTCGCGTAGGCGAA
GATGTATCAATAGAAGTAAAAGCATCTAATCCCTTCTCGTACTTAGTATACGTAATTGTAGGACGAGGTAACATTTTG
CAGATGAGAAACATACCCTTGCCTAATCCGCAGAACTTTTACACGATAACTTTTAAAAGCACCTTCGAGATGATACCT
AACGCGCATGTATTTGTTTACTATGTTGACAACGGTGATCTAAAATTTCAAGAAATTGGCGTTAAAATTCAACCGGAA
TTTGAGAATAAGATTGAAGTAACTGGCCCTAGCCAAGCGAAGCCTGGCCAAGAAGTTACTTTGAATGTTAAAACCGAT
CCGAATTCATATGTTGGTCTATTGGGGGTGGATCAAAGCGTCTTATTATTGAAGTCTGGCAACGATTTGCATCGCCAT
GCTATACTCAATGATTTAAAACTTTACAAAAATTGGGAAGAGAATGAATGGCCAAGAAGCGTTTACAAAGTTCCTGGG
AAAAAATCTGGTTTGCTGATAATGACCAATTCCCATTATCCCTATGAAGTTTTGTACATGATGGCTTCACCTACTATG
TACCAATTACGTGCTCCTGTTGCTATGCCGACTTCAATCAATGCGTTTTCAATACCTGCACCCGTCTCTTTGCCCGCA
CCTTTAATGCGTTCAGCGGAACCGCAAAGGATTCGAAAAGACTTTGCTGAAGTTTGGTTGTTCGATAGTTTGGATGAT
AACAATGAAACTGGACACTTGACACTGATCAAATCTCTGCCTGATACAATAACTTCTTGGGTAATCAGCGCTTTTGCG
GTGAACGAGAAAACTGGTCTAGGTATGACCGAGAATCCGTTCAAAATTAACGTATTTCAACCATTCTTCATCGATGTA
AATTTGCCATATTCTGTAAAAAGGGGCGAAGTCATTGCCATACCAGTTATTATTTTCAACTATATGGATAAAACATTG
GATGCAGAAATCACCATGGACAATACTGACAAGGAGTACGATTTCACCGAAGTGTCCAATGAAATTGAGGAATCTATT
CTGAGCGCGCAAAAGAGGATAAAACGGGTATCGGTGCCTCCGAATAGCGGAGAAAGCGTATCTTTTATGATACGTCCA
ACCGTAGTAGCTGATATAGAATTGAAAATTAACGCTATATCTCAGTTGGCAGGGGACGCTATACATAAAAAACTAAAA

GTAGAAGCTGAAGGTGTCACCCAATACAAAAATCAAGCTCTTTTCTTAAATTTGGAGAAGCCACAGGAAGCTTCACTA
GAAGTCGCTATACCCACGGAAGCTGTTAAAGACTCGGAATATGTGGAATTCTCTGTTGTTGGTGATCTCTTGGGTCCC
ACTGTCAAAAATCTTAATGAGCTTGTACGTAAGCCCTACGGTTGTGGTGAGCAGAATATGGTTAATTTCGTGCCCAAC
ATATTGGTCTTACATTACTTGGAAGCCATGCAAATTGATATGCCAAATGTAGCGAGCAAAGCTAAAAACTTCTTAGAA
ATTGGTTATCAACGTGAGTTGACCTATAAGCACAAAAACGGCGCATATAGCGCTTTTGGCGAAGATAGAAGCACACCG
AATACTTGGTTAACCGCTTATGTGGCTCGTTCCTTTATCCAGGCATCTAAATACACGACCATTGATGAAAATGTTATC
CAACAAGCTTTCGAGTTCCTAATTGCTAACCAGGAAAAGAGCGGGCAATTTAAGCAAACTGGTCATCTCTTCAGTCCA
ACTCATCAGAATGATGTTGGATTCAATGCTTATGTGCTGTTGGCCTTCCTAGAAAGTGAAAAATACGCGGAGGTTTAT
CAAAAGCAGATTGAAAAATGCCTCGAATACGTAGTAAGCCAGCTGGAGAATGAAAAGGATTCTTACGCTTTATCTATA
GCTGCAACTGCTTTGCATAAAGCTAATCATAAGGCTGCGGGTGGAGTGCTGGAAAAGCTACAGGCACAAGCCAAGGAA
GAAAACGGTTTCAAAAGGTGGACTAGTGCGAACCATAATGATATTGAAATCACTGCCTATGCGTTGCAAACTTTGGTT
GACATGGAATCCACTAATCAAATTTTACCAATTATCAAGTGGTTAATCGGTCAACGTAATAGCAATGGTGGTTTCGAC
TCTACCCAAGACACTGTAGTAGGTCTTGAGGCACTTATTAAATTCTCTAAGAAATTTTCAATTACCGGGAACAGCATA
ATGTCGATAACATTCAAAGCTTTTGATGATGGAAAGAAGGAACTTAGCCAGCATAGATTTGAGGTGAACAAAGACAAC
TCATTGGTCTTACAAACGCACGTGTTACCGAAATCCACACGTTCAATAAGTTTGGAAGCTGATGGTGTTGGTTCTTCG
TTAATTCAACTTTCGTATCAATACAACTTGGCAACCAAAGACGATAGACCTGGATTCAAAGTAGACATTAAGCCGAAA
ATTTTGCCATCGCAACAGCTGCAGATTAATATTTGCGCAAATTATCAACCGGCTGTTGACGATGAAATCAAGGAATCC
AATATGGCTGTCATGGAAGTTGCTTTGCCATCTGGTTATATCGCCGACAATGAGAAATTTAATGATATACTTGCTGTC
GAGCGTGTTCAACGCGTCGATACCGAAAACTCTAATACAAAAGTGATCGTCTACTTTGATGGCTTAGTGGAAGGTGAA
CAAGTATGTGTTACTATCCTAGGCAGACAAAGCTTTTGCTGTTGCTaagcagaaaccagttcccgtcactctgtacga
ctactacaatagcatatatcgtggaaccgaatattatcaaattgaatcttcactgtgcgatatttgtgagggaagtga
ttgtggaactgaatgtaaatgaaattatactaatattataatttgtgtccacttaataaataaagttccaaattgaag
